# Mycobiomes of Six Lichen Species from the Russian Subarctic: A Culture-Independent Analysis and Cultivation Study

**DOI:** 10.3390/jof11120848

**Published:** 2025-11-29

**Authors:** Armen Hakobjanyan, Alexey Melekhin, Marina Sukhacheva, Alexey Beletsky, Timofey Pankratov

**Affiliations:** 1 S.N. Winogradsky Institute of Microbiology, Research Centre of Biotechnology of RAS, 119071 Moscow, Russia; hakobjanyanarmen0@gmail.com; 2 Faculty of Biology and Biotechnology, National Research University “Higher School of Economics”, 101000 Moscow, Russia; 3 N.A. Avrorin Polar-Alpine Botanical Garden Institute, 184209 Apatity, Russia; melihen@yandex.ru; 4 Tobolsk Complex Scientific Station of the Ural Branch of RAS, 626152 Tobolsk, Russia; 5 Skryabin Institute of Bioengineering, Research Centre of Biotechnology of RAS, 119071 Moscow, Russia; moldiag@biengi.ac.ru (M.S.); mortu@yandex.ru (A.B.)

**Keywords:** lichens, mycobiome, fungal diversity, *Cetraria*, *Nephromopsis*, *Stereocaulon*, *Cladonia*, cryptic species

## Abstract

Lichens are defined as holobionts, whose thalli are known to contain a significant diversity of bacteria, fungi, protozoa, and viruses. Research into the presence of these organisms in lichens remains limited. Therefore, assessing the diversity of fungi in different species of lichen remains a relevant task. In this study, we analysed the taxonomic composition of the mycobiome of six lichen species from northern Russia. To achieve this, we employed high-throughput sequencing and cultivation methods using a modified nutrient medium. The study obtained data on the dominance of fungi from the classes *Dothideomycetes*, *Eutypomycetes*, *Leotiomycetes* and *Tremellomycetes* in the lichen samples studied. We found that the most common taxa among the lichen species studied were lichenicolous or parasitic fungi belonging to the genera *Athelia*, *Epithamnolia* and *Cladosporium*. The diversity of OTUs in *Nephromopsis nivalis* thalli that were processed using an abrasive to remove epiphytic fungi was found to be 30–50% lower than in intact thalli. Our findings suggest that the characteristics of the lichen species and its environment within the biocenosis can influence the diversity and abundance of fungi in thalli. Ninety-two fungal cultures were obtained and identified at various phylogenetic levels. Six strains were identified that presumably belong to new families within the orders *Lecanorales*, *Tremellales*, *Septobasidiales* and *Myriangiales*. We discovered that modifying cultivation methods can hasten the quest for novel, hitherto unexplored strains of lichenophilic fungi.

## 1. Introduction

Lichens are an example of a symbiotic association between fungi and phototrophic organisms capable of surviving and reproducing in extreme conditions. These complex organisms are characterised by an absence of dense barrier coverings, such as plant bark or animal skin, to protect them from the environment; their bodies are open systems that are only weakly isolated from the external environment. The diversity of lichens and their involvement in food, topical and phoretic interactions reflects their success in mountainous, tundra and humid tropical forest ecosystems. Surrounded by soil, plants and animals, lichens accumulate significant quantities of microorganisms in their thalli [1,2,3,4], which are associated with the mycobiont and photobiont through various forms of interaction, ranging from neutralism to parasitism. A significant and diverse component of lichen thalli is non-lichenized fungi, which, according to the latest findings, can be divided into two main groups [5]: (1) epiphytes, which inhabit or are temporarily present on the surface of thalli in the form of reproductive structures, mycelium or individual cells, and (2) endophytes, whose mycelium or cells are integrated into the intercellular spaces of mycobiont hyphae and photobiont cells. The first group includes both transient and permanently inhabiting species. The second group comprises parasites of mycobionts or algae, as well as a group of endophytes known as asymptomatic fungi. These fungi do not cause morphological or physiological changes in thalli and are often undetectable by classical visual examination of the thallus, even under a microscope [6]. Hafelnner [7] defined three subgroups of fungi that inhabit lichens. The first subgroup, lichenicolous fungi, are specific to lichens and can be identified by their morphological characteristics. This group includes approximately 1800 described species belonging to the Ascomycota and Basidiomycota phyla [8,9,10,11]. The second subgroup comprises endolichenic fungi (endophytes of lichens that are hidden or difficult to distinguish based on morphological characteristics, arising from primary non-lichenised lines of *Dothideomycetes*, *Eurotiomycetes*, *Leotiomycetes* and *Sordariomycetes* [12,13,14,15]. These fungi are still poorly understood and difficult to diagnose using standard microscopic methods; their study requires a combination of molecular and culture-dependent methods. The third subgroup comprises lichen epiphytes: fungi that are usually lichenised and grow on lichen thalli. They are more commonly found among *Lecanoromycetes* [16,17]. Additionally, yeast forms can be found among epiphytes, localised among the surface structures of thalli [18,19]. Non-lichenised fungi in lichens, especially asymptomatic endolichenics, are of interest due to their significant diversity [20]. Following the introduction of high-throughput sequencing methods, a similar issue emerged to that encountered with bacterial microbiomes: a significant proportion of the microbial diversity pool consisted of previously uncultivated fungal lineages [21]. Using a limited range of traditional culture media such as wort agar, starch-dextrose agar and Sabouraud’s medium did not facilitate the isolation of new fungal taxa from lichens, leaving a significant amount of diversity unexplored. Departing from this practice by using nutrient media with low sugar and starch content, along with the addition of polyols, vitamins, and trace elements, and increasing incubation times at lower temperatures, led to the discovery of new species and genera of fungi [19,22,23,24,25].

Of the many lichen species, several groups are ecologically significant and have economic and medical importance. These groups are capable of forming lichen mats and accumulating large amounts of biomass in temperate and cold regions. Examples of these ubiquitous species include *Cetraria islandica*, *Cladonia arbuscula*, *Cl. rangiferina*, *Cladonia stellaris*, *Cl. uncialis*, *Nephromopsis nivalis* and species from the genera *Alectoria*, *Stereocaulon* and *Umbilicaria*.

Despite the fact that *Cetraria islandica* is widely distributed and economically important, the diversity of microorganisms in this lichen species has been poorly studied. Most of the existing literature focuses on lichenicolous fungi [26,27,28] and bacterial diversity in the thalli of these lichens [29,30]. Studies of the mycobiome of this lichen species using both cultural and non-cultural methods have probably not been conducted before. More data is available for *Nephromopsis nivalis* (*Flavocetraria nivalis*), as Zhang et al. published the results of 454 pyrosequencing of seven lichen samples collected in the Svalbard archipelago in 2015 [31]. These lichens were dominated by representatives of the orders *Helotiales*, *Saccharomycetales* and unknown ascomycetes. Studies of the mycobiomes of *Cladonia stellaris* and *Cl. arbuscula* have also been sporadic [31,32], although a fair amount of data is available on lichenicolous fungi [33]. Lichens of the *Cl. arbuscula* species [31] were dominated by representatives of the *Capnodiales* order (*Dothideomycetes*) and the *Helotiales* order (*Leotiomycetes*), as well as a significant proportion of ascomycetes of unknown origin. According to Shishido [32], *Cl. stellaris* and *Cl. arbuscula* were dominated by representatives of the *Eurotiales* and *Onygenales* orders (*Eurotiomycetes* class). These differences are evident as a result of samples being collected in different biotopes within different climatic zones. Among the lichenicolous fungi of lichens in the genus *Stereocaulon* are species such as *Arthonia stereocaulina* and *Opegrapha stereocaulicola* (order *Arthoniales*, class *Arthoniomycetes*), *Rhymbocarpus stereocaulorum* (order *Cyttariales*, class *Leotiomycetes*) and *Roselliniella stereocaulorum* (order *Hypocreales*), and others [34,35]. No metabarcoding data for the mycobiomes of species of this genus were found.

To understand the strategy of lichen mycobiome formation, it is crucial to study the taxonomic composition of fungi in identical or closely related lichen species found in different, distant ecosystems under similar or dissimilar climate conditions. This approach can successfully assess the presence of random fungal species, as well as those that have become permanent components of thalli due to selection processes. In other words, this will enable us to determine which types of fungi occur randomly in lichen mycobiomes and which are chosen and established as permanent components.

Vančurová et al. [36] carried out such work on algae and lichens of the genus *Stereocaulon*. This study demonstrated that the relationship between the mycobiont and the photobiont is more significant than geographical and climatic conditions. It was also noted that, regardless of climatic or geographical conditions, the mycobiont *Stereocaulon* selects a partner from three genera of algae.

An analysis of mycobiome similarity in two lichen species [25] revealed that the proportion of *Eurotiomycetes*, *Dothideomycetes* and *Sordariomycetes* representatives in the fungal communities of *Rhizoplaca melanophthalma* and *Tephromela atra* lichens depended on geographical location. Some genera were also found in samples collected in distant geographical locations. Comparative analysis of species from Turkey and South Korea revealed common fungal taxa in two lichen genera, *Peltigera* and *Parmelia* [37]. These common taxa were unidentified representatives of *Chaetothyriales*, *Dothideomycetes* and Fungi. Overall, few studies have been devoted to the comparative analysis of geographically distant lichen mycobiomes.

This study aimed to determine the taxonomic structure of the mycobiomes of six lichen species located 800 km apart, but within similar climatic zones. We also aimed to compare metabarcoding data with the results of cultural methods of analysing fungal diversity in thalli of the same species and other species of lichen collected at the same time in winter. In order to understand the differences between the same species in different locations, we selected two identical species (*Cetraria islandica* and *Nephromopsis nivalis*, also known as *Flavocetraria nivalis*) and two pairs of closely related lichen species within the same genus. To establish possible connections between mycobiomes within a single location, we selected different genera and species of lichen.

## 2. Materials and Methods

### 2.1. Study Sites and Sample Collection

Samples were collected in December 2024 from two geographically distinct locations: the Khibiny Mountains (vicinity of Kirovsk) and the area around of Naryan-Mar city (Table 1). Samples from the first site were collected in the Botanical Cirque of the Khibiny Mountains, at an altitude of between 450 and 580 m above sea level. The samples were collected in the following locations: (1) at the edge of the elfin birch forest, from under a layer of snow (20 cm) on the soil (*Cladonia stellaris* (Opiz) Pouzar & Vĕzda); (2) in the tundra, on rocky scree, shallowly under snow, from a stone (*Cetraria islandica* (L.) Ach., *Nephromopsis nivalis* (L.) Divakar, A.Crespo & Lumbsch); (3) in the tundra, on a section of a snow-free cliff, from a stone (*Stereocaulon vesuvianum* Pers.). The plant communities in these biotopes were dominated by angiosperms such as *Arctostaphylos uva-ursi* (L.) Spreng., *Dryas octopetala* L., *Empetrum nigrum* L., *Kalmia procumbens* (L.) Gift, Kron & P. F. Stevens, *Oxytropis sordida* (Willd.) Pers., *Oxyria digyna* (L.) Hill and *Phyllodoce caerulea* (L.) Bab. Mosses were represented by *Andreaea rupestris* Hedw., *Niphotrichum canescens* (Hedw.) Bedn.-Ochyra & Ochyra, *Racomitrium lanuginosum* (Hedw.) Brid., *Pleurozium schreberi* (Willd. ex Brid.) Mitt. and *Sanionia uncinata* (Hedw.) Loeske.

Samples were collected in duplicate from each location, with point coordinates taken to an accuracy of three metres. Using sterile gloves, snow was carefully removed from around the lichens and the thalli were aseptically placed in sterilised, double-layered paper bags for transportation. The first batch of specimens was shipped immediately (wet, with ice and snow in the bags) by post to the laboratory. They dried during shipping and were free from mould damage. The second set of specimens were dried at room temperature for several hours before shipping.

One sample was taken from each microbiological specimen, all of which are stored in the KPABG herbarium. Identification was carried out using the standard method [38] at the PABGI KSC RAS instrument base.

Samples from the second site (Naryan-Mar) were collected from a 10 × 10 m test plot located in a depression in the microrelief, which had large areas of blown sand. The altitude here is 10 m above sea level. This area was dominated by a dwarf shrub-lichen community consisting mainly of *Cladonia stellaris*. The plant communities in these biotopes were dominated by the following species: *Betula nana* L., *Larix sibirica* Ledeb., *Vaccinium vitis-idaea* L., *Hylocomium splendens* (Hedw.) Schimp. and *Empetrum nigrum* L. Lichen samples were collected from larches along the edge of the plot on a raised area of terrain within a larch-birch dwarf shrub-moss community, where *Vaccinium vitis-idaea* and *Hylocomium splendens* were predominant in the ground cover. The lichen samples from Naryan-Mar were processed and identified in the same way as the samples from the Khibiny Mountains. After receiving the specimens were stored at −20 °C until further processing. Lichen taxon names are given according to Westberg et al. [39].

### 2.2. Preparing Samples for Inoculation onto Agarised Culture Media

**Figure 1:**
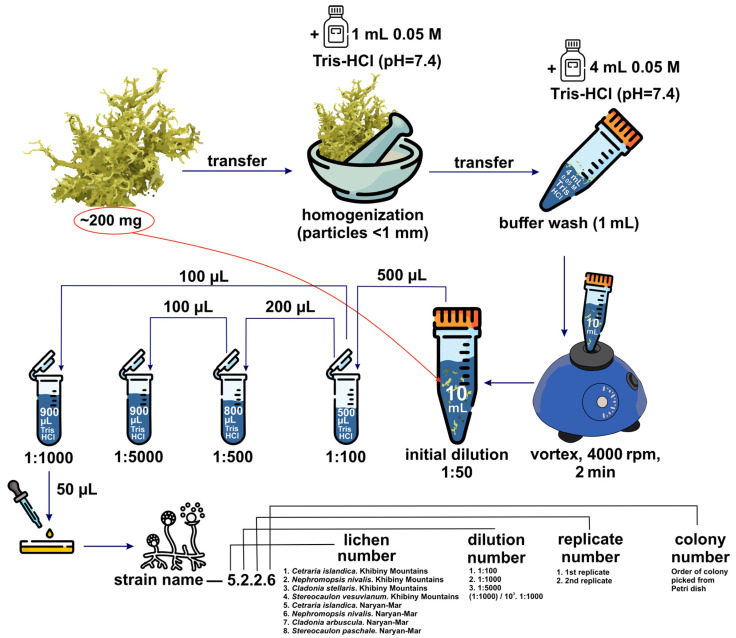
A schematic representation of the dilution procedure and strain labelling. Inoculation was performed using a Drigalski spatula on an agar nutrient medium with the following composition (g/L): glucose—4; mannitol—1; xylose—1; tryptone—1; yeast extract—1; ammonium sulphate—0.1; potassium phosphate monobasic—0.1; magnesium sulphate—0.05; calcium nitrate—0.025; chloramphenicol—0.1; a complex of trace elements and vitamins; and agar—15 g. For each dilution variant, 50 µL of the suspension was applied to each Petri dish (two replicates per condition). The inoculated plates were incubated at 16 °C under a 12 h light/12 h dark cycle for two months. Cultures were monitored every two weeks. As a result, a series of isolated colonies were obtained and subsequently used to establish pure cultures.

A portion of the lichen thallus was placed in a mortar and moistened with 1 mL of 0.05 M Tris-HCl buffer (pH 7.4). The material was ground to a homogeneous state, with particle sizes smaller than 1 mm. The resulting homogenate was transferred into a sterile 15 mL centrifuge tube prefilled with 4 mL of 0.05 M Tris-HCl buffer. Residual material was rinsed from the mortar using an additional 1 mL of the same buffer, which was also added to the tube. Subsequently, 4 mL more of the buffer was added, resulting in a final volume of 10 mL plus the biomass. The suspension was vortexed at 4000 rpm for 2 min. Figure 1 shows the sample preparation procedure.

### 2.3. Preparation of Samples, DNA Extraction, Amplification, and Sequencing

#### 2.3.1. DNA Extraction from Lichen Thalli

Total DNA was extracted from lichen thalli using the FASTDNA™ SPIN Kit for Soil (MP Biomedicals, Yantai, Shandong, China) with modifications to the initial sample preparation steps. Approximately 200 mg of lichen thallus was finely chopped with sterile scissors into fragments 0.5–2 mm in size. The material was transferred into a sterile porcelain mortar, supplemented with 200 μL of Tris-HCl buffer (pH 8.0), and ground into a homogeneous paste-like suspension. The resulting homogenate was transferred into a lysing matrix tube containing beads. Next, 500 μL of PBS buffer (137 mM NaCl, 2.7 mM KCl, 10 mM Na_2_HPO_4_, and 1.8 mM KH_2_PO_4_; pH 7.4) was added to the tube, followed by centrifugation at 10,000 rpm for 1 min (MiniSpin, Eppendorf, Hamburg, Germany). The tubes were then frozen at −15 to −20 °C for 30–60 min. Subsequently, 122 μL of MT buffer (provided in the kit) and 200 μL of PBS were added. Mechanical disruption was performed using a FastPrep-24 instrument (MP Biomedicals) in two 40 s cycles at 6 m/s, with manual mixing between cycles to ensure even distribution of beads. The contents of the tubes were then transferred using a sterile metal spatula into a 5 mL sterile syringe preloaded with sterile glass wool, which was compressed with the syringe plunger. Care was taken to prevent large beads (≥1.4 mm in diameter) from entering the syringe. The liquid phase was transferred into a sterile 2 µL microcentrifuge tube by pressing down on the plunger. The plunger was removed, and an additional 270 μL of PBS was added to the syringe to extract the remaining liquid, which was also collected into the same tube. All tubes were volume-equalised using PBS buffer. Samples were centrifuged for 5 min at 14,000 rpm, and the supernatant was transferred to new sterile 2 mL microcentrifuge tubes. DNA extraction was then continued according to the manufacturer’s protocol for the FASTDNA SPIN Kit for Soil.

#### 2.3.2. DNA Extraction from Pure Cultures

Genomic DNA was extracted from yeast fungal biomass using a modified CTAB-based protocol. Fungal biomass was transferred to 2 mL microcentrifuge tubes, followed by the addition of 1 mL TE buffer and 20 μL of lysozyme solution (20 mg/mL). Samples were incubated in a thermo-shaker (TS-100C SC-24C, BioSan, Riga, Latvia) at 37 °C and 800 rpm for 30 min. Tubes were centrifuged for 2 min at 14,000 rpm, and the supernatant was discarded. The pellet was resuspended in 500 μL TES buffer and incubated at 60 °C for 30 min, followed by freezing at −20 °C. After thawing, 140 μL of 5 M NaCl and 65 μL of preheated 10% CTAB solution were added to the sample. The mixture was incubated at 65 °C for 10 min. Subsequently, 705 μL of a chloroform:isoamyl alcohol mixture (24:1) was added, and the sample was vortexed (LV-1006, Elmi, Latvia) at 3000 rpm for 1 min until flocculent material disappeared. Tubes were then incubated at −15 °C for 15 min and centrifuged for 10 min at 14,000 rpm. The upper (aqueous) phase was transferred to a new tube, and 225 μL of 3 M sodium acetate was added, followed by brief vortexing. After incubation at −15 °C for 15 min, the samples were centrifuged for 5 min at 14,000 rpm. The supernatant was transferred to a 1.5 mL tube, and DNA was precipitated by adding 0.55 volumes of isopropanol. The mixture was incubated at −20 °C for 20–30 min to allow DNA aggregation, then centrifuged for 5 min at 14,000 rpm. The supernatant was removed, and the pellet was washed twice with 1 mL of cold 70% ethanol. After each wash, the sample was briefly vortexed and centrifuged for 2 min at 14,000 rpm. Ethanol was carefully removed, and the pellet was air-dried under a UV lamp for approximately 10 min. DNA was then resuspended in 50 μL of TE buffer. Samples were incubated in a thermo-shaker (TS-100 SC-20, BioSan, Latvia) at 37 °C for 30 min. If complete dissolution of the DNA was not achieved, tubes were left overnight at 4 °C and re-incubated. Extracted DNA was stored at −20 °C. For filamentous fungi, the initial steps differed: a 5 × 5 mm agar block containing fungal mycelium was excised using a sterile scalpel. The biomass was transferred into a 2 mL tube containing 500 μL of TE buffer, homogenised with a sterile spatula, and supplemented with 500 μL of distilled water. Lysozyme solution (20 μL, 20 mg/mL) was added, and samples were incubated at 37 °C for 2 h at 800 rpm. The pellet obtained after centrifugation (2 min, 14,000 rpm) was resuspended in 500 μL of TES buffer, and 6 μL of proteinase K was added to inactivate nucleases. Next, 40 mg of sterile powdered glass was added, and the mixture was gently stirred by hand. Tubes were placed on a cold rack and frozen at −15 °C for 15 min. Ice and biomass disruption was carried out using a rotary drill with a custom grinding tip, taking care not to touch the walls of the tube to avoid microplastic contamination. Samples were then incubated in a thermo-shaker at 60 °C and 800 rpm for 30 min. From the addition of 140 μL of 5 M NaCl and CTAB onward, the protocol followed that described for yeast-like fungi.

#### 2.3.3. Processing of *Nephromopsis nivalis* Thalli to Remove Epiphytic Propagules

To remove fungal propagules from the thallus surface, thallus fragments were vigorously shaken in 1 mL of pre-cooled (5 °C) TRIS buffer (pH 6.5), with the addition of 100 mg of sterile silica gel for chromatography with a particle size of 60 μm, as well as 100 mg of silica gel with a particle size of 100–150 μm. This process was carried out in 2 mL test tubes. A Fast Prep homogeniser (MP Biomedicals, Santa Ana, CA, USA) was used. The shaking mode was set to 40 s at a speed of 6 m/s, repeated twice. After processing in the homogeniser, the biomass was placed in a syringe fitted with a sterile glass fibre filter. The buffer residues were removed using a plunger and the biomass washed three times with 20 mL of sterile water. The resulting biomass was then used for the total DNA extraction process described above.

#### 2.3.4. The Process of Obtaining Amplicons for the Analysis of Pure Fungal Cultures via PCR

**Figure 2:**
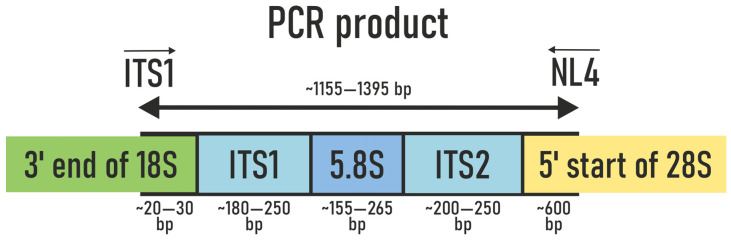
Schematic representation of the PCR product. The arrows show the direction of DNA chain synthesis. ITS1 is the forward primer and NL4 is the reverse primer.

To identify the obtained fungal strains, PCR amplification was performed targeting a genomic region that partially includes the 18S rRNA gene (small subunit), the complete ITS region, 5.8S rRNA, ITS2, and a part of the 28S rRNA gene (large subunit). Two primers were used: ITS1F (5′-CTTGGTCATTTAGAGGAAGTA) and NL4R (5′-GGTCCGTGTTTCAAGACGG) [40,41] (Figure 2). The expected size of the amplified fragment was approximately 1300 base pairs (bp).

#### 2.3.5. Electrophoresis of Genomic DNA and PCR Products

The presence of DNA and the length of PCR fragments were assessed by horizontal electrophoresis in agarose gel. Electrophoresis was performed at 96 V for 60 min. Visualisation of the results was carried out using the Molecular Imager Gel Doc XR documentation system (Bio-Rad, Hercules, CA, USA). The resulting images were recorded and processed using the Quantity One software v. 4.6.5 (Bio-Rad, USA).

#### 2.3.6. Preparing PCR Products for Sanger Sequencing

Sequencing was performed using an automated capillary sequencer 3730 DNA Analyzer (Applied Biosystems, Foster City, CA, USA) at the Bioengineering Shared Research Centre at the Federal Research Centre for Biotechnology of the Russian Academy of Sciences in Moscow, Russia. Samples for Sanger sequencing were prepared as follows: in a 200 μL microtube, 5 μL of purified PCR product (template) was mixed with 1 μL of a primer at a concentration of 1.6 pmol/μL. Each PCR product was thus submitted for sequencing with both forward and reverse primers as two separate reactions.

#### 2.3.7. Sanger Sequencing

The obtained electropherograms in “ab1” format were processed using MEGA version X [42]. To generate high-quality sequences, electropherograms were trimmed; in the case of sequences obtained using the NL4 primer, reverse complementary sequences were generated. Multiple sequence alignment and contig assembly were performed using Unipro UGENE v. 52.0 [43]. The resulting contigs were analysed using the Basic Local Alignment Search Tool (BLAST) [44]. The obtained sequences, along with reference sequences of type strains from the NCBI and MycoBank databases (BLAST; MycoBank), were aligned using MEGA X. Taxonomic identification of each strain was based on the percentage of sequence identity (Table 2), with sequence coverage exceeding 90% in most cases.

#### 2.3.8. NGS Sequencing and Identification of OTUs

PCR amplification of ITS fragments was carried out using the universal primers ITS-F (5′-CYHVGTYATTTAGAGGAASTAA-3′) and ITS-R (5′-GCTGCGTTCTTCATCGHTGB-3′) [46]. PCR fragments were barcoded using the Nextera XT Index Kit v.2 (Illumina, San Diego, CA, USA). The PCR fragments were purified using Agencourt AMPure beads (Beckman Coulter, Brea, CA, USA) and quantitated using the Qubit dsDNA HS Assay Kit (Invitrogen, Carlsbad, CA, USA). Then, all of the amplicons were pooled together in equimolar amounts and sequenced on the Illumina MiSeq (2 × 300 nt paired-end reads). Overlapping paired Illumina reads were merged using FLASH v1.2.11. Usearch v.11 commands were used to cluster merged reads into OTUs at 97% identity threshold. Low quality reads, chimeric sequences and singletons were excluded from the OTUs during the analysis. To estimate the frequencies of OTUs in each sample reads were mapped to OTUs using Usearch at 97% identity threshold. Taxonomy of OTUs was predicted using UNITE ITS reference database v.10 and Sintax classification algorithm in Vsearch v2.28.1.

BLAST algorithms were used in the MycoBank and NSBI databases to identify OTE sequences, as had been done for pure cultures before.

### 2.4. Calculation of Colony-Forming Units (CFU) per Gram of Dry Sample

The calculation was performed using the following formula:

(1) $$CFU/g=\frac{N*F}{V*m}$$

where *N* is the number of colonies grown on the plate (units); *F* is the dilution factor (reciprocal of the dilution); *V* is the volume of the suspension plated onto the Petri dish (mL); *m* is the mass of the dry sample (g).

### 2.5. Determination of Alpha Diversity Indices

We used the following indices to assess the alpha diversity of the fungal community in lichens: species richness (the number of unique species), Shannon index [47] (higher values indicate greater community diversity; values close to 0 indicate a community dominated by a single species, while high values range from 2 to 4).

(2) $$H^{\prime }=-\sum_{i=1}^{S}p_{i}\times ln(p_{i})$$

where *H′* is the Shannon index; *S* is the total number of species; *p_i_* is the proportion of the *i*-th species in the total community (the number of isolates of one species divided by the total number of isolates, i.e., the frequency or probability of occurrence of species *i*); *ln* is the natural logarithm.

The Simpson index [48] measures dominance within the community (values close to 1 indicate dominance by a single species).

(3) $$D=-\sum_{i=1}^{S}n_{i}(n_{i}-1)/N(N-1)$$

where *D* is the Simpson index, representing the probability that two randomly selected individuals belong to the same species; *S* is the total number of species; *n_i_* is the number of individuals of species *i* in the sample; *N* is the total number of individuals of all species in the sample; *n_i_*(*n_i_* − 1) is the number of ordered pairs of different individuals of the same species *i* (sampling without replacement); *N*(*N* − 1) is the total number of ordered pairs of any two individuals in the entire sample.

### 2.6. Light Microscopy

Micrographs were taken using an Axio Imager 2 light microscope (Carl-Zeiss, Jena, Germany).

### 2.7. Statistical Analysis and Visualisation

Statistical analysis and visualisation were performed using the MS Excel 2010 and Orange V.3.39.0 software packages.

### 2.8. Deposition of Sequences of Pure Cultures and Illumina NGS Sequences

The OTU sequences obtained by NGS profiling have been deposited in GenBank under the accession numbers PX406301-PX406485 and PX502199-PX502209. The 18S rRNA (partial), ITS1, 5.8S rRNA, ITS2 and 28S rRNA (partial) gene sequences are deposited in GenBank under the following accession numbers: PX352608–PX352694.

## 3. Results

### 3.1. Identification of Mycobionts

In addition to the traditional identification method using diagnostic keys, ITS1 gene sequences were analysed using high-throughput sequencing. The sequences obtained were analysed using BLAST algorithms (NCBI). The results are presented in Table 3.

Metabarcoding, which uses primers to amplify the ITS1 region of rRNA, confirmed that the samples belonged to the expected species. However, the *S. vesuvianum* and *S. paschale* samples were found to contain *S. alpinum* sequences. The *Cl. arbuscula* lichen samples contained sequences of *Cl. submitis* and *Cl. uncialis*. Nevertheless, morphological analysis and the presence of dominant OTUs belonging to species identified based on morphological and biochemical characteristics confirm that these samples belong to *S. vesuvianum*, *S. paschale* and *Cl. arbuscula*.

### 3.2. OTU Analysis

All samples were analysed in duplicate for each site, with the results then compiled. The number of initial reads varied depending on the sample (Table 4). For samples of the same species collected at the same locus, the number of initial reads could differ by more than twofold; for example, the *C. islandica* samples from Naryan-Mar showed this variation. The number of unique OTUs identified in replicates of a single sample also varied. The highest number of unique OTUs was identified in *Cladonia* and *S. paschale* samples, and the lowest in *N. nivalis* samples that had been processed using abrasive materials. The percentage of sequencing reads was acceptable for processing the sequencing results further and ranged from 88% to 96%.

### 3.3. Analysis of Mycobiome Structure Using High-Throughput Sequencing

We analysed eight paired samples (16 samples in total) of lichens, as well as four processed samples of *N. nivalis* located in the Khibiny Mountains and Naryan-Mar. The four samples were processed using an abrasive material to obtain impurity-free DNA (see the ‘Materials and Methods’ Section 2). As the total proportion of OTUs unrelated to mycobiont OTUs was low (Table 5), the data set was normalised by taking the sum of all OTUs unrelated to mycobionts as 100%. Non-major sequences related to *Lecanoromycetes* and other species of lichenised fungi were also excluded. Repeats were averaged. Statistical error graphs are presented in Figure S1.

**Figure 3:**
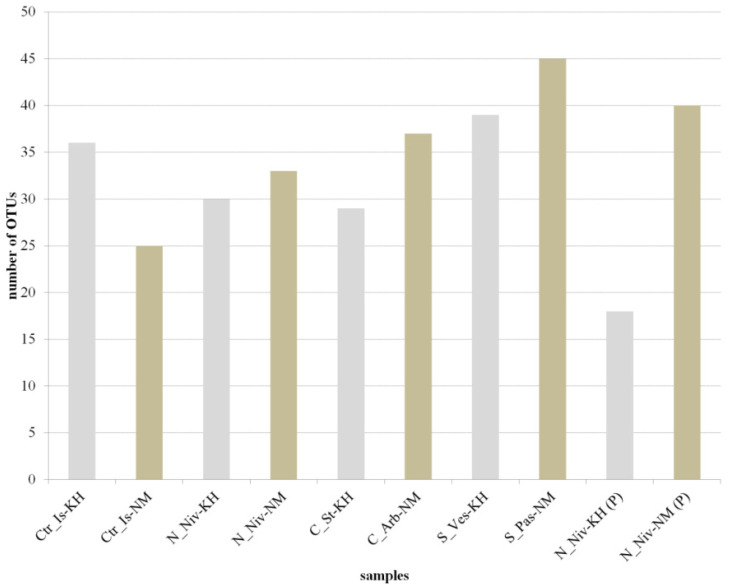
Number of OTUs obtained after normalisation and filtering with a threshold of 0.49% for ten lichen samples. Ctr_Is: *Cetraria islandica*; N_Niv: *Nephromopsis nivalis*; C-St: *Cladonia stellaris*; C_Arb: *Cladonia arbuscula*; S_Ves: *Stereocaulon vesuvianum*; S_Pas: *Stereocaulon paschale*; KH: Khibiny; NM: Naryan-Mar; (P): processed samples. The samples from Khibiny are indicated by the grey bars, and the green bars show the samples from Naryan-Mar.

Following normalisation, it was decided to exclude those minor OTUs that accounted for less than 0.49% (±0.04) of all OTUs in the pool and that did not belong to the mycobiont. Consequently, we obtained sets of OTUs that dominated the composition of the mycobiomes of the studied lichens (Figure 3). The taxonomic affiliation and the relative contribution of OTUs to the mycobiome communities of the studied lichens are shown in Supplementary Table S3.

The highest number of dominant OTUs was observed in samples of *S. paschale*, *S. vesuvianum* and *N. nivalis*. The fewest dominant OTUs were found in *C. islandica* (NM) and *Cl. stellaris* samples.

### 3.4. Alpha Diversity in Mycobiomes

**Figure 4:**
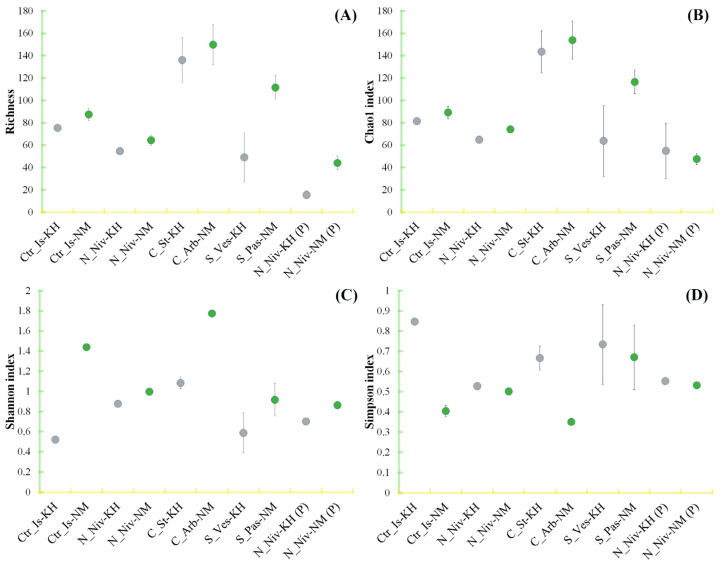
Alpha diversity: richness (**A**), Chao1 index (**B**), Shannon (**C**) and Simpson (**D**) coefficients for the fungal communities detected via OTU analysis of all sequences retrieved from the corresponding samples. Data from two replicates of each sample were analysed for lichen communities from Khibiny (KH) and Naryan-Mar (NM), as well as for the following species: *Cetraria islandica* (Ctr_Is), *Nephromopsis nivalis* (N_Niv), *Cladonia stellaris* (C_St), *Cladonia arbuscula* (C_Arb), *Stereocaulon vesuvianum* (S_Ves) and *Stereocaulon paschale* (S_Pas), and for processed samples (P). The grey dots show samples taken in Khibiny, while the green dots show samples taken in Naryan-Mar.

**Figure 5:**
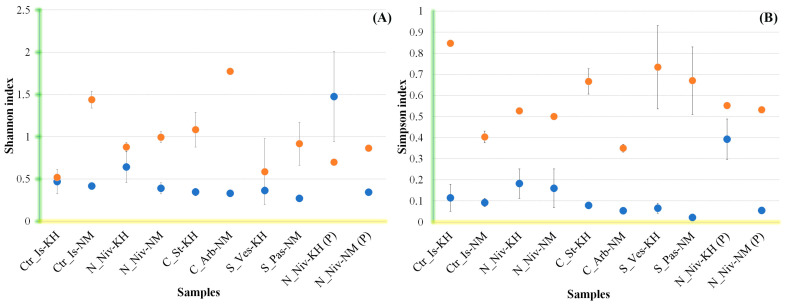
Plots of the Shannon (**A**) and Simpson (**B**) indices for fungal communities detected via OTU analysis of all sequences (orange dots) and sequences with an abundance greater than 0.49% (blue dots) are shown, as retrieved from the corresponding samples. The data used for the analysis, which were obtained from two replicates for each sample, are given for the lichen communities from Khibiny (KH) and Naryan-Mar (NM), as well as for the species *Cetraria islandica* (Ctr_Is), *Nephromopsis nivalis* (N_Niv), *Cladonia stellaris* (C_St), *Cladonia arbuscula* (C_Arb), *Stereocaulon vesuvianum* (S_Ves) and *Stereocaulon paschale* (S_Pas), and for the processed samples (P).

Alpha diversity was calculated based on statistical data obtained from the entire OTU array for each sample, with non-fungal OTUs excluded (Figure 4). The same calculation was performed on the data array without mycobiont sequences (Figure 5).

Both species of *Cladonia* exhibited the greatest species diversity, with a Chao1 index above 140. For the other samples, the index was below 120 (Figure 4B). At the same time, species diversity was higher in lichen samples from Naryan-Mar than in samples from the Khibiny Mountains. There were no significant differences in species richness indices between *Cetraria* and *Nephromopsis* lichens, either within species or between territories. The most significant difference in Chao1 indices was found between *Stereocaulon* lichen samples, reaching a relative value of 53. Richness values correlated positively with Chao1 index values, except for two processed samples of *N. nivalis* (Figure 4A).

The Shannon diversity index indicates the uniformity with which different taxa are distributed in a sample. At higher values, it reflects the importance of taxa that are present in low numbers but that may be ecologically significant. The most distinct difference in this indicator was observed between lichens from two locations (Figure 4C). Notably, intact thalli of *N. nivalis* and thalli treated with an abrasive suspension did not differ significantly in this respect, whereas the richness indicator did differ significantly between these samples, particularly for samples from the Khibiny Mountains.

The Simpson index indirectly measures dominance rather than diversity. It calculates the probability that two individuals selected at random from the same sample belong to the same species. Therefore, when the index is high, the probability of several species dominating is also high. As expected, this index increases slightly in samples treated with abrasive, since the removal of epiphytic fungi increases the proportion of dominant endophytes in the total mycobiome (Figure 4D). The dramatic difference between *C. islandica* from Khibiny and Naryan-Mar is less clear. The decrease in the weight of dominant species in the sample from Naryan-Mar implies a high invasive load on the thallus from the ecosystem. Similar differences are also evident in two species of *Cladonia*.

As the overall assessment of OTU diversity in samples is based on a comparison of the full range of amplicons (OTU reads), diversity indicators are distorted by the abundance of DNA from the dominant fungus (the mycobiont). To determine the diversity of non-mycobiont OTU sequences, these sequences were filtered out and the remaining sample analysed using Shannon and Simpson indices. The results of this analysis are shown in Figure 5.

Overall, after removing the mycobiont OTU and other minor OTUs (less than 0.49%), the values of both indices decreased, indicating a lower level of diversity within the fixed range of contribution values from 0.49% to 100%. At the same time, we found a low level of difference in the Shannon index and a very significant difference in the Simpson index in the samples of *C. islandica* (KH) and *S. vesuvianum*. Therefore, for these species, the diversity of the entire sample is equivalent to that of a narrow range of dominant species. In other words, there are few minor OTUs and they do not significantly contribute to the overall diversity pool. In other cases, however, minor OTUs contribute significantly to overall diversity, and among them there are groups that are emerging as potential dominants. This assumption is confirmed by the results of the analysis of *N. nivalis* (KH) samples treated with abrasive material. The Shannon index increased significantly, exceeding the values of the index for the total OTU pool.

Depending on the site, the Simpson index in the processed samples increases or decreases, indicating a change in the diversity of dominant fungal species.

### 3.5. Analysis of the Taxonomic Affiliation and Relative Contribution of the Dominant OTUs

**Figure 6:**
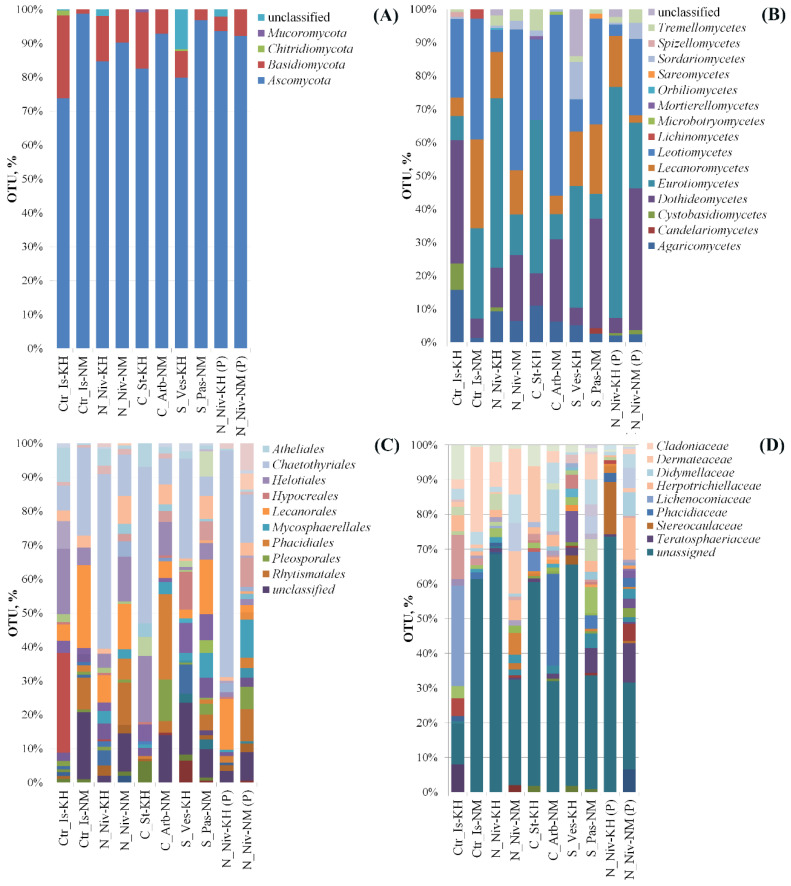
Relative abundance plots of fungi for divisions (**A**), classes (**B**), orders (**C**) and families (**D**) in lichen communities from Khibiny (KH) and Naryan-Mar (NM) for the species *Cetraria islandica* (Ctr_Is), *Nephromopsis nivalis* (N_Niv), *Cladonia stellaris* (C_St), *Cladonia arbuscula* (C_Arb) and *Stereocaulon paschale* (S_Pas). Abundance data are shown as a percentage. ‘Unclassified’ includes all other taxa not identified among the OTU sequences. Only the orders and families whose OTUs are above 10% in at least one sample are shown. The taxonomic affiliation and the relative contribution of OTUs to the mycobiome communities of the studied lichens are shown in Supplementary Table S3.

We analysed the relative contribution and taxonomic affiliation of OTUs selected based on dominance (Figure 3). The results of this analysis are shown in Figure 6.

Ascomycetes predominated in all samples. The proportion of *Basidiomycetes* was higher in samples from the Khibiny Mountains. Only the *C. islandica* and *S. vesuvianum* samples from the Khibiny Mountains contained OTUs classified as *Chytridomycetes*. OTUs that could not be identified as belonging to any known type or class of fungi were also found in the *C. islandica*, *N. nivalis* and *S. vesuvianum* samples from the Khibiny Mountains. These were OTUs No. 623, 624 and 229 in the *S. vesuvianum* samples and accounted for 9.9% of dominant OTUs in total. According to the MycoBank alignment results, OTU 229 belongs to the phylum *Cryptomycota* (formerly *Rozellomycota*), which was first validated by Doweld in 2013 (https://www.indexfungorum.org/names/NamesRecord.asp?RecordID=550328 (accessed on 27 November 2025) [49].

In the *N. nivalis* (KH) sample, OTU No. 369 accounted for 1.6% of the total. This amplicon is also present in the OTU library of the processed *N. nivalis* sample, at a proportion of 2.2%. The clone has the greatest similarity (at 30% coverage) to the soil clone KY687698 from Sweden [50]. The phylogeny of these sequences is shown in Figure S2. The closest sequences were selected based on similarity, although in most cases the coverage level was low. A minor *Mucoromycota* clone was found only in unprocessed *Cl. stellaris* (KH) samples. It was one of the representatives of the *Mortierella* genus.

Three classes of ascomycetes were dominant in the samples: *Dothideomycetes*, *Eurotiomycetes* and *Leotiomycetes* (Figure 6B). This is consistent with previous data on the dominant classes in lichen mycobiome composition [31,51]. The classes *Agaricomycetes* and *Lecanoromycetes* were subdominant. The remaining classes were represented by minor OTUs. In abrasive-processed *N. nivalis* (KH) samples, the proportion of *Eurotiomycetes* OTUs increased significantly while that of *Dothideomycetes* and *Agaricomycetes* decreased. The proportion of *Lecanoromycetes* OTUs remained unchanged. The result was different in the processed *N. nivalis* (NM) sample. Here, the proportion of *Dothideomycetes* and *Eurotiomycetes* OTUs increased, while the number of *Leotiomycetes* and *Lecanoromycetes* OTUs decreased. Thus, in both variants, only the proportion of *Eurotiomycetes* increased upon abrasive treatment. This class of ascomycetes includes many fungi that specialise in parasitism. Lichens often become their hosts [31].

The *Sordariomycetes* class and unclassified OTUs significantly contribute to the composition of the *S. vesuvianum* mycobiome.

All samples exhibited significant OTU diversity belonging to various orders (Figure 6C). Representatives of 49 orders were identified in the total OTU pool. Clones related to the orders *Helotiales*, *Chaetothyriales* and *Atheliales* were detected at varying quantities in all samples. Representatives of the orders *Pleosporales*, *Mycosphaerellales*, *Lecanorales* and *Cladosporiales*, as well as sequences unidentified to order level, were usually found in most samples (usually with the exception of one or two). The remaining orders were less represented. In *N. nivalis* (KH) lichens, both unprocessed and abraded thalli were dominated by *Chaetothyriales* representatives. This order also dominates the communities of *Cl. stellaris* (KH) and *S. vesuvianum* (KH).

A specific marker for *C. islandica* from the Khibiny Mountains is the order *Lichenoconiales*, as no OTEs associated with this taxonomic group were found in other samples. Conversely, representatives of the order *Rhytismatales* are clear indicators of the biotope. Only samples from Naryan-Mar contained OTEs associated with this order. These OTUs account for between 3% and 12% of all OTUs with a percentage contribution of more than 0.49%. Additionally, in abrasive-processed samples of *N. nivalis* (NM), the proportion of these OTUs decreases (from 12% to 9%), though it remains relatively high. Given that many representatives of this order are plant parasites, the data on their significant contribution to the mycobiomes of Naryan-Mar lichens are consistent with the results of alpha diversity analysis. The orders *Xylariales*, *Sakaguchiales*, *Phaeothecales*, *Orbiliales*, *Mycocaliciales*, *Lecideales*, *Filobasidiales*, *Cystobasidiales*, *Candelariales* and *Baeomycetales* can be classified as rare in the analysed samples, with their OTUs occurring sporadically. In other words, representatives of these orders are not closely associated with lichens and are most likely random species that have been transmitted from the niches of surrounding ecosystems. This is indirectly evidenced by the more significant enrichment of these OTUs in *N. nivalis* samples from Naryan-Mar.

At the family level, the sample data from the study does not show any regularity (Figure 6D). The largest number of identified families was found in *C. islandica* (KH) samples, followed by *N. nivalis* (NM), *Cl. arbuscula* (NM) and *S. paschale* (NM) samples. Between 59% and 73% of OTUs in the *C. islandica* (KH), *N. nivalis* (KH), *Cl. stellaris* (KH) and *S. vesuvianum* (KH) samples could not be identified at the family level using the ITS1 region. These sequences most likely represent new taxa at the family or genus level. OTUs associated with *Atheliaceae* were present in all samples, as were representatives of *Cladosporiaceae*, *Herpotrichiellaceae*, and *Teratosphaeriaceae*.

At the genus and species level, a large proportion of OTUs could not be accurately identified, either because they represent new taxa or because they cannot be identified based on the ITS1 region alone. The genera and species that were accurately identified are presented in Table S1. All samples contain OTUs associated with the genera *Cladosporium*, *Epithamnolia* and *Fibularhizoctonia* (anamorph *Athelia*). The genus *Cladosporium* is represented by the species *C. hillianum*, *C. cladosporioides*, *C. angustiherbarum* and *C. herbarum*. *E. rangiferinae* and *E. xanthoriae* were present in *N. nivalis*, *Cl. stellaris* and *S. paschale*. The genus *Athelia* was not represented by any known species. Most of the genera identified at genus level are plant parasites, lichens, or lichenicolous species. However, some are represented by saprotrophs, including *Hypholoma*, *Trichoderma*, *Cortinarius*, and *Penicillium*.

### 3.6. The Relationship Between the Presence of Fungal Taxa and Lichen Species, and Their Geographical Location

**Figure 7:**
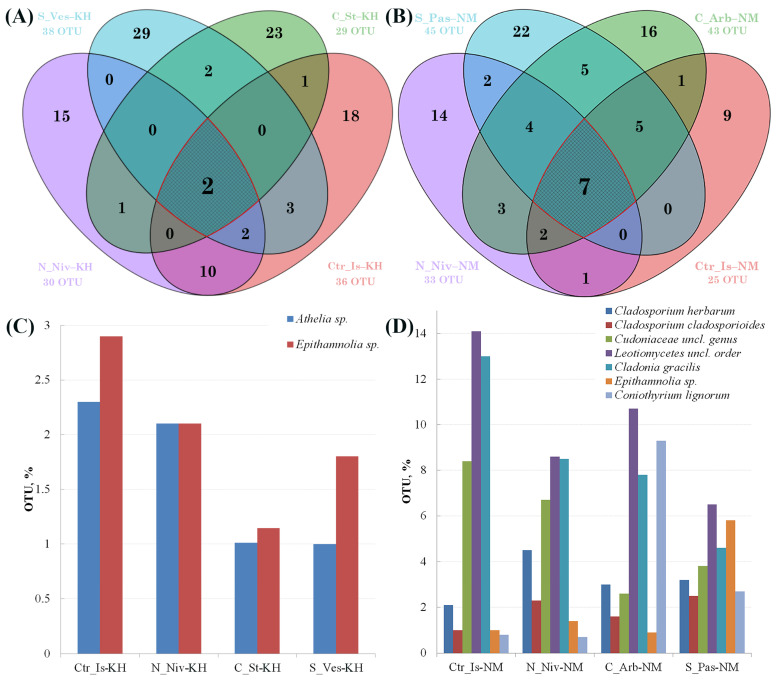
Venn diagrams showing the overlap in OTUs between species of Khibiny lichens (**A**) and Naryan-Mar lichens (**B**). Shared OTUs are shown in a larger font size. The taxonomic affiliation and relative abundance of OTUs are shown in the histograms for lichens from Khibiny (**C**) and Naryan-Mar (**D**). The taxonomic affiliation and the relative contribution of OTUs to the mycobiome communities of the studied lichens are shown in Supplementary Table S3.

To determine which taxa are common to different species within a single location or genus of lichens separated by 800 km, we conducted an analysis of the logical relationships between several sets or groups. Figure 7 shows Venn diagrams illustrating the similarities and differences in the mycobiome compositions of lichens at two sites.

The mycobiomes of all Khibiny samples contain only two OTUs that can be classified as new species of the genera *Athelia* and *Epithamnolia* (Figure 7A,C). Both taxa predominate in *C. islandica* and *N. nivalis*. Members of these genera are primarily parasitic and lichenicolous fungi of lichens [52,53]. The genus *Athelia* also contains species that are pathogenic to insects and plants [54,55]. Based on our results, these species appear to be non-specific parasites of lichens, capable of infecting various host species. Clearly, the biotope and surrounding ecosystem strongly influence mycobiome composition. In lichens from Naryan-Mar, we observed a different composition of common taxa. Here, uncultivated fungi belonging to the class *Leotiomycetes*, as well as *Cladonia gracilis*, dominate. A significant proportion of the representatives of the class *Leotiomycetes* are phytopathogenic fungi that cause severe damage to plants.

The presence of the *Cladonia* OTU is difficult to explain, given that the samples were carefully prepared for analysis and the presence of foreign thalli was excluded. Furthermore, they are present in all eight samples from the four species. During development, it is likely that mycobionts in close contact with each other in one locus are able to germinate and be present in the thalli of morphologically similar species. However, the possibility that *Cl. gracilis* soredia were mechanically transmitted to the thalli of other lichens cannot be ruled out. Two species of *Cladosporium*, *Epithamnolia* sp. and *Coniothyrium lignorum*, were also present in all four lichen species from Naryan-Mar. All of these species are facultative or obligate parasites. Lichens from Naryan-Mar generally show closer ecological connections, as they share more fungal taxa with each other than with samples from the Khibiny Mountains.

Shared taxa for respective species and closely related species are shown in Figure S3. *Epitamnolia* sp. (OTU29), *Cladosporium cladosporoides* (OTU16) and *Athelia* sp. (OTUs 19 and 20) were present in all sample pairs from different loci, except for the processed *N. nivalis* samples. Five common OTUs were found in *S. vesuvianum* and *S. paschale* samples, including the aforementioned OTUs and genera (OTU14: *Cladosporium herbarum*, OTU20, and OTU29). *Stereocaulon* lichen samples differ from the others in that they contain the common OTUs 62 and 88 (*Venturia* sp. and *Leotiaceae* sp., respectively). The highest number of common OTUs (seven) was observed in *N. nivalis* samples.

### 3.7. Search for Epiphytic and Endophytic Fungal Groups in Nephromopsis nivalis

This lichen species has flat thalli without tubular structures, a smooth surface and a well-developed cortex, which makes it convenient for studying the ratio of epiphytic and endophytic microorganisms. Two populations of *N. nivalis* were selected from habitats differing in altitude, temperature and precipitation. Parallel samples of thalli were subjected to abrasive processing (see Section 2), after which DNA was isolated from the thoroughly washed thalli.

**Figure 8:**
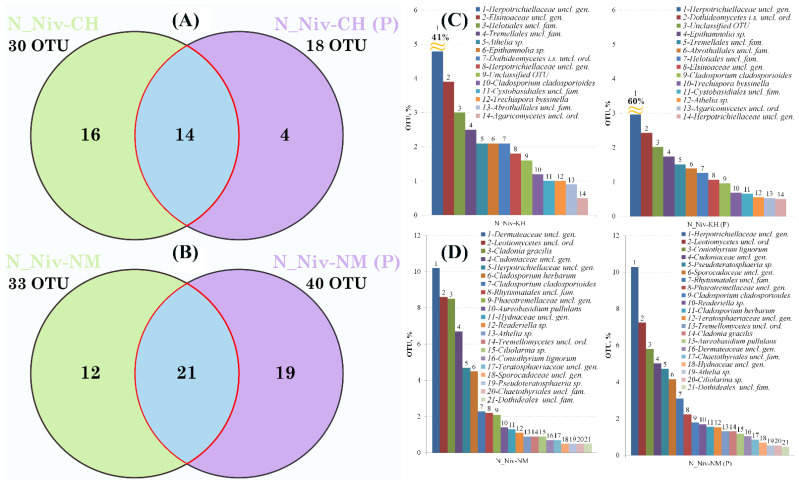
Venn diagrams showing the number of shared OTUs between processed and unprocessed *Nephromopsis nivalis* samples from Khibiny (**A**) and Naryan-Mar (**B**). Taxonomic affiliation and relative abundance of OTUs are shown in the histograms for samples from Khibiny (**C**) and Naryan-Mar (**D**). The shares of individual OTUs are shown in the histograms for intact (**left**) and processed (**right**) thallus samples. The taxonomic affiliation and the relative contribution of OTUs to the mycobiome communities of the studied lichens are shown in Supplementary Table S3.

The results of the OTE ratio analysis are shown in Figure 8.

In this case, Venn diagrams clearly illustrate the number of fungal OTUs that remained after the thallus surface was processed. While we cannot claim to have removed all epiphytic colonies or mycelium due to the absence of sterility control procedures on the thallus surface or microscopy controls, the results showed a decrease in the number of OTU taxa contributing more than 0.49% in the Khibiny population and an increase in samples from Naryan-Mar. It is interesting that the proportion of *Cl. gracilis* decreased in the processed sample from Naryan-Mar (Figure 8D #3 and #14). It is possible that this species was present on the surface of the *N. nivalis* thallus in the form of powdery soralia but was tightly integrated into its cortical layer.

The result showing an increase in the number of OTUs in the washed sample from Naryan-Mar seems somewhat paradoxical. However, it should be noted that the increase in the number of OTUs reflects an increase in the proportion of those OTUs. These OTUs were minor in the structure of the microbiomes of the untreated samples. It would therefore be more accurate to present the proportion of OTUs out of the total in the untreated and treated samples. For thalli from Khibiny, the proportion of total OTUs in the unprocessed thallus is 47%, compared to 78% in the processed thallus. For thalli from Naryan-Mar, the proportions are 70% and 52.5%, respectively. In other words, in samples from Naryan-Mar, the proportion of epiphytic dominants is higher than that of forms hidden inside the thallus. When these dominant forms are removed from the surface, the community’s structure changes and the dominant OTUs become those that were minor before processing.

Thus, the predominant sequences in the “core” of the mycobiome were those represented by lichenicolous and parasitic taxa from the classes *Dothideomycetes* and *Agaricomycetes*. When comparing the Venn diagrams obtained for unprocessed and processed samples from different locations (Figure S3), we see that the number of common OTUs decreases after processing from seven to four common OTUs. The main “endophytic” taxa become *Cladosporium* and *Athelia*, along with *Stereocaulon* and a representative of an unidentified genus in the family *Herpotrichiellaceae*.

### 3.8. Analysis of the Diversity of Cultivated Fungi

We used the standard cultivation method in Petri dishes containing an agarised nutrient medium whose composition was selected to meet the specific needs of lichen-forming fungi [19].

**Figure 9:**
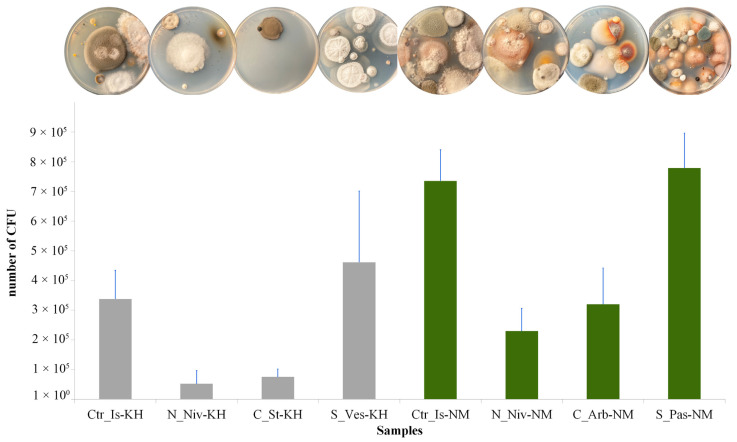
The number of colony-forming units (CFUs) of fungi counted in eight lichen samples per gram of air-dried thallus. The grey columns show the values for the Khibiny samples, and the green ones show the values for the Naryan-Mar samples. Statistical processing was performed based on four replications (*p* < 0.05).

The highest abundance of CFUs (colony-forming units) was diagnosed in two lichen samples from Naryan-Mar: *C. islandica* and *S. paschale* (Figure 9). The lowest number of fungal CFUs were isolated from *N. nivalis* and *Cl. stellaris* in the Khibiny Mountains.

Most isolates exhibited standard mycelial growth and formed large-diameter colonies. Dark-coloured and melanised colonies were generally small and compact, as were yeast colonies.

The species richness index, calculated based on the phylogenetic analysis of 92 isolates, was highest in *C. islandica* samples from Khibiny (10) and Naryan-Mar (15), and in *Cl. arbuscula* (NM) samples (Table 6).

Overall, the species diversity is higher in the Naryan-Mar samples. It was impossible to calculate the species diversity index for the *Cl. stellaris* (KH) samples due to the small number of CFUs. *C. islandica* (NM) and *Cl. arbuscula* (NM) samples demonstrate the maximum Shannon index. This is consistent with the diversity data obtained using the NGS method. The proximity of the Shannon and Simpson indices in *N. nivalis* (KH) samples indicates a small number of species, with one taxon dominating. This is corroborated by the data in Table S2.

Thirteen strains were obtained from *C. islandica* (KH) samples, 28 from *C. islandica* (NM), seven from *N. nivalis* (KH), two from *Cl. stellaris*, 16 from *S. vesuvianum*, two from *N. nivalis* (NM), 12 from *Cl. arbuscula* and 12 from *S. paschale* (Table S2). Thus, the largest number of isolates were obtained from *C. islandica* and *S. vesuvianum* samples. Various ascomycete and basidiomycete fungi were isolated from most lichen samples. However, from *S. vesuvianum*, we mainly obtained strains that were close to, or identical to, *Tolypocladium inflatum*, which is the asexual form of *Cordyceps subsessilis* [56]. Two other isolates belonged to new genera in the *Fayodiaceae* and *Hyphodiscaceae* families.

We analysed the logical relationships between sets of strains identified at genus level. Venn diagrams revealed that none of the fungal genera were common to all four lichens from Khibiny. Similarly, we found no shared genera among the four lichen species from Naryan-Mar (Figure S4). However, *C. islandica* (KH) and *N. nivalis* (KH) shared three genera: *Hypholoma*, *Leptosporomyces* and *Lichenoconium*. *C. islandica* (NM) and *Cl. arbuscula* (NM) shared two genera: *Occultifur* and *Oidiodendron*. *C. islandica* (NM) contained two genera that were also present in *S. paschale*: *Phoma* and *Sydowia*. Overall, the results demonstrated a closer relationship between lichen species from Naryan-Mar.

**Figure 10:**
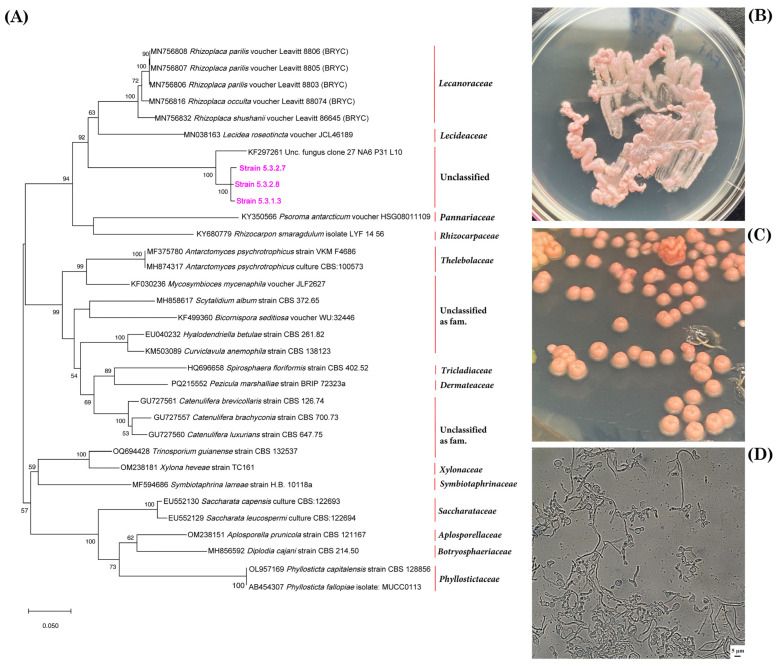
Phylogenetic position of three fungal strains (5.3.2.7, 5.3.2.8 and 5.3.1.3) isolated from the lichen *C. islandica* (NM) in relation to closely related taxa in the order *Lecanorales* (**A**); growth of strain 5.3.2.7 on FA+ agar medium (**B**); colonies of strain 5.3.2.7 on FA+ medium (**C**); morphology of the mycelium of strain 5.3.2.7 (**D**). Magnification: 600×; scale bar: 5 μm.

Analysis of the ITS1-5.8S-ITS2 gene sequences revealed that 21 of the isolated cultures belonged to new genera and that another six could be assigned to new families. Five of the six strains were isolated from *C. islandica* (both from KH and NM), while only one was isolated from *N. nivalis* (from NM). Three strains (5.3.2.7, 5.3.2.8 and 5.3.1.3) are phylogenetically identical and are likely to be clones retrieved from a single population (see Figure 10A). They belong to the *Lecanoromycetes* order, *Lecanorales*. These strains are most closely related to *Rhizoplaca* and *Lecidea*, and they may represent a new family within this order (Figure 10B). This is also indicated by the morphology of the colonies and cells, which resembles that of the lichen genera *Rhizoplaca* and *Lecidea* (Figure 10C).

**Figure 11:**
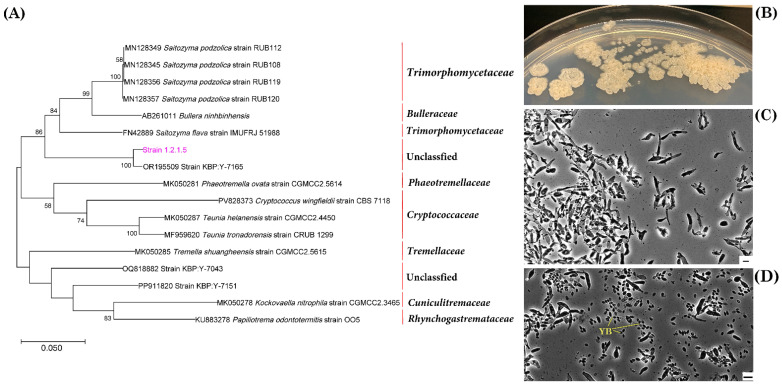
The phylogenetic position of strain 1.2.1.5, which was isolated from the lichen *C. islandica* (KH), is shown in relation to related taxa in the order *Tremellales* (**A**). The growth of strain 1.2.1.5 on FA+ agar medium is shown in (**B**). The morphology of strain 1.2.1.5 cells is shown in (**C**). The comparative morphology of young buds (YB) of mature cells is shown in (**D**). Magnification: 600×; scale bar: 5 μm.

**Figure 12:**
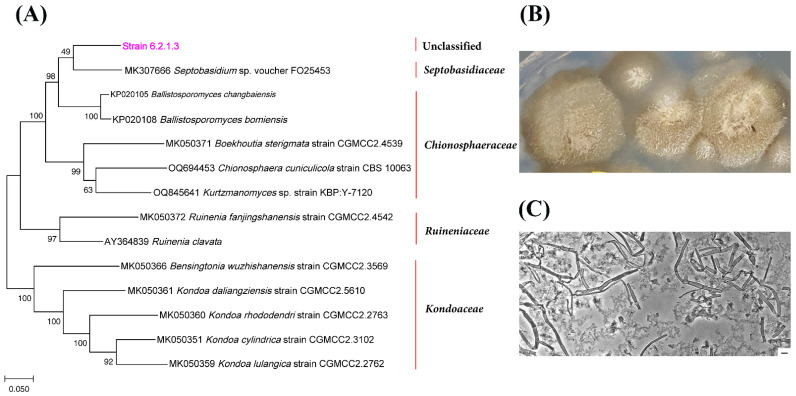
The phylogenetic position of strain 6.2.1.3, which was isolated from the lichen *N. nivalis* (NM), is shown in relation to related taxa in the order *Septobasidiales* (**A**). The growth of strain 6.2.1.3 on FA+ agar medium is shown in (**B**), and the morphology of the mycelium of strain 6.2.1.3 is shown in (**C**). Magnification: 600×; scale bar: 5 μm.

**Figure 13:**
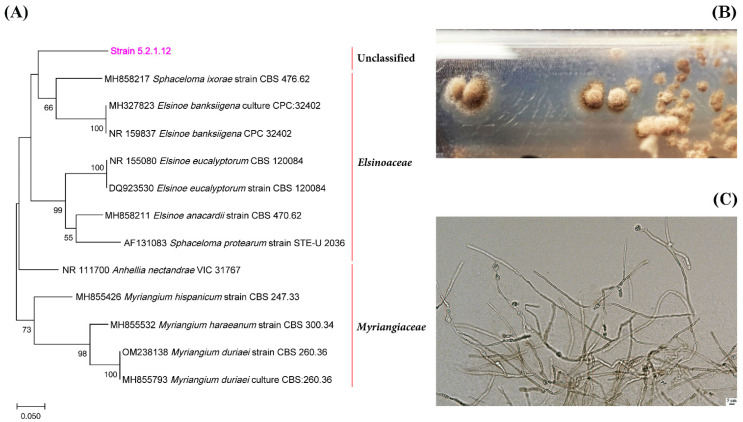
The phylogenetic position of strain 5.2.1.12, which was isolated from the lichen *C. islandica* (NM), is shown in relation to related taxa in the order *Myriangiales* (**A**). The growth of strain 5.2.1.12 on FA+ agar medium is shown in (**B**). The morphology of strain 1.2.1.5 mycelium is shown in (**C**) at a magnification of 600×. The scale bar is 5 μm.

The ITS1/ITS2 gene sequences of strain 1.2.1.5 clustered together with representatives of the order *Tremellales* (Figure 11A). The closest match was strain KBP Y-7165 (98.62% similarity; GenBank number OR195509), which was previously isolated from *Stereocaulon* sp. (unpublished). The closest type strain was *Tremella shuangheensis* strain CGMCC2.5615 (85.49%; GenBank number MK050285) [57]. Morphologically, these are yeast cells and the colonies are dry, compact and yellowish-beige (Figure 11B,C). Strain 6.2.1.3, which was isolated from *N. nivalis*, was phylogenetically closest to representatives of the families *Septobasidiaceae* and *Chionosphaeraceae*, particularly the genera *Septobasidium* and *Ballistosporomyces* (Figure 12A). However, it formed a separate branch, with 87.61% similarity to the strain with which it was most closely related, *Septobasidium* sp. (MK307666). Strain 5.2.1.12, isolated from *C. islandica* (NM), exhibited the greatest phylogenetic similarity to *Myriangium duriaei* (MH855793), at 83.18%, and *Anhellia nectandrae* (NR111700), at 84.14%, thus demonstrating a relationship with representatives of the order *Myriangiales* (Figure 13A). This strain forms compact, dark brown colonies with mycelial morphology (Figure 13B,C).

Most of the isolated cultures were identified at species and genus level. Common species included *Tolypocladium inflatum*, *Sydowia* sp., *Cladophialophora minutissima*, *Aureobasidium pullulans*, *Cladosporium ossifragi*, *Phoma herbarum* and *Penicillium lividum*, among others (Table S2). A significant proportion of the species and genera identified were known plant pathogens or parasites of lichens and fungi. Surprisingly, the samples of *Stereocaulon vesuvianum* were dominated by species of the *Tolypocladium* genus, which are known to be parasites of *Coleoptera* and mycophagous [56]. By contrast, *S. paschale* was dominated by the phytopathogens *Phoma herbarum* and *Sydowia* sp. [58,59], the saprotroph *Penicillium lividum* and the entomophagous fungus *Beauveria brongniartii* [60].

## 4. Discussion

During this study, we discovered that fungal sequences not associated with the lichen mycobiont accounted for 3–22% of all identified OTUs. This percentage varies depending on the lichen species and the sample used for DNA or CFU isolation. The lowest values for this indicator were found in *S. vesuvianum* and the highest in *Cl. stellaris* and *Cl. arbuscula*. Therefore, the total proportion of epiphytic and endophytic fungi is less than a quarter of the total OTU pool. In abrasive-processed samples of *N. nivalis*, the proportion of OTUs belonging to non-mycobionts decreases two to threefold, indicating the dominance of epiphytic fungi in lichen thalli. We also found that the proportion of non-mycobiont OTUs is higher in lichens from Naryan-Mar. Subsequent analysis confirmed that samples from Naryan-Mar generally contain more OTUs and cultivated species. This difference is related to the characteristics of the biotopes: samples from Khibiny were mainly collected from tundra areas with minimal tree and shrub vegetation, whereas samples from Naryan-Mar were collected from tundra areas near coniferous forests and lakes. The influence of biotope characteristics is an important reason for differences in plant mycobiome composition [61], and the same is true for lichen microbiome composition [62].

Analysis of the most abundant OTUs, accounting for over 0.49% of non-mycobiont OTUs, revealed that the fewest such sequences were found in Khibiny samples. Among the species studied, *Stereocaulon* samples exhibited the highest value of this indicator, likely due to the morphology of their thalli (the presence of phyllocladia and pseudocyphellae), which accumulate significant dust particles from the air. Little is known about the accumulation of dust particles by lichen thalli, but it is established that thalli actively absorb heavy metals and other pollutants from the air [63]. The greater the thallus’s specific surface area, the greater the probability of accumulating particles transported by air masses.

The highest OTU species diversity index was found in the mycobiomes of *Cladonia*, and the lowest was found in samples of *N. nivalis*. The same is true of the cultivated forms of fungi identified in these samples. It can be assumed that the morphology of the thallus influences the extent to which it becomes contaminated with dust and aerosol particles, as well as cells of fungi, algae and bacteria. The greater the thallus’s specific surface area, the more likely it is that particles will adhere to it. This assumption requires further experimental verification in terms of lichen research. Among the studied samples, *N. nivalis* has the smallest specific surface area and a developed cortex layer. This prevents particles from being retained on the surface and reduces the likelihood of their introduction into the underlying layers of the thallus. The Shannon index reflects the uniformity of the distribution of different taxa in a sample and varies depending on the lichen’s location rather than its species affiliation. This once again emphasises the importance of environmental influences on the composition of lichen microbial communities [62]. At the same time, a higher Shannon index value suggests that minor taxa significantly contribute to the structure of the mycobiome. The highest Chao1 index values are characteristic of the two studied species of *Cladonia*. Interestingly, fewer cultivable fungal forms were isolated from these samples than from others. This is most likely due to their inability to grow on the applied culture media and under the cultivation conditions. Well-adapted forms, such as *Cladosporium*, *Penicillium* and various yeast fungi, can easily be detected in lichen thalli [64,65]. Conversely, the slow-growing forms identified by us and other researchers are rarely isolated from lichens due to the use of traditional nutrient media and cultivation times and conditions. We found that discarding OTUs with a lower weight (less than 0.49%) in the mycobiome composition resulted in altered Shannon and Simpson indices in almost all samples, with the exception of *N. nivalis* from Khibiny. Surprisingly, the Shannon index increased in the sample treated with abrasive, indicating an increase in the proportion of OTUs that were minor in the analysis of the entire sample. These OTUs likely constitute the uncultivable pool of endolichenic organisms [66]. This is confirmed by the fact that the Simpson index remains virtually unchanged, indicating that a pool of dominant taxa remains in addition to minor taxa and that these taxa acquire greater weight in the community. The taxonomic structure of communities at different hierarchical levels generally resembles that previously described [2,6,13,15,18,19,21,31,67]. However, at the division level, differences emerge between samples from different locations. For instance, in samples from the Khibiny Mountains, we identified a greater number of OTUs affiliated with the *Basidiomycota* division. At class level, the dominant OTUs were sequences belonging to the classes *Dothideomycetes*, *Eurotiomycetes* and *Leotiomycetes*, which is consistent with previous data obtained for these and other lichen species [13,31,37,68]. The presence of *Lecanoromycetes* sequences in almost all samples is an interesting finding. These are predominantly lichenised ascomycetes and their presence in lichens as part of minor fungal groups rather than as mycobionts has been described previously [69].

Overall, this study confirmed the predominance of fungi belonging to the classes *Dothideomycetes*, *Eurotiomycetes*, *Leotiomycetes* and *Tremellomycetes* in lichens, as previously reported [13,31,37,67,68]. The fungi that represent these classes are often mycophiles and parasitise both fungi and lichens, as well as plants [3,70,71,72]. Many of them are plant endophytes or insect parasites [55,60,72].

Our data showed that the composition of the lichen mycobiome depends significantly on the biotope in which the thallus forms. In mono-sinusias and in the absence of higher plants (such as in tundra or rocky mountain areas), the diversity of fungi in thalli may be low and limited to lichenicolous species and obligate parasites. However, in the presence of higher plants and mosses, as well as in complex, multi-species lichen communities, the diversity of fungi in thalli increases due to the integration of phytopathogenic species and species associated with insects. The consistent presence of fungi belonging to the genera *Cladosporium*, *Phoma* and *Penicillium* [20,64,65,66] in lichens of different species and climatic zones may confirm their ability to grow and survive in adverse conditions across a wide range of ecological niches. However, their confirmed presence in the endolichenic mycobiome may also indicate their specificity to lichens as a habitat [20,65,66]. We discovered that certain fungal orders can serve as markers for both lichen species and biotopes.

Our study of mycobiome similarities has led us to conclude that fungi that are obligate to lichens are represented by mycoparasites (e.g., *Tolypocladium*) and specific lichenicolous fungi (e.g., *Epithamnolia*). At the same time, each lichen species has its own unique set of fungi, as confirmed by a recent publication [73]. The new isolates that we have identified are previously uncultivated representatives of the orders *Lecanorales*, *Septobasidiales*, *Tremellales* and *Myriangiales*. These are slow-growing forms that are adapted to low temperatures and low concentrations of sugar in the nutrient medium. Using poorer nutrient media in combination with the addition of polyols and polysaccharides against a background of low glucose or sucrose concentrations may result in the isolation of more new taxa from lichens, as well as a reassessment of our understanding of the diversity of previously uncultivated fungi inhabiting lichens.

## 5. Conclusions

In this study, we demonstrated that lichens harbour a significant variety of fungal species, primarily belonging to the *Ascomycota* and *Basidiomycota* divisions. The most abundant fungal groups are lichenicolous fungi, mycoparasites and phytopathogens belonging to the classes *Dothideomycetes*, *Eurotiomycetes*, *Leotiomycetes* and *Tremellomycetes*. Most of the taxa identified are specific to certain lichen species, and the most common were lichen parasites and lichenicolous fungi with unknown ecological strategies. The diversity and abundance of fungi in lichen thalli depend on both species’ affiliation and habitat conditions. A higher invasive load from the biocenosis may result in thalli containing a greater number of colony-forming units, leading to higher taxonomic diversity. The taxonomic diversity of fungi in lichens may also depend on their location within the thallus. Removing epiphytic microorganisms can significantly decrease the diversity and abundance of fungi and alter the taxonomic structure of the mycobiome. Cultivated forms of fungi that grow well on traditional nutrient media were characterised by low diversity and represented well-known, widespread species and genera. However, using a depleted nutrient medium led to the isolation of new, previously uncultivated taxa. In the future, the routine isolation of previously uncultivated fungi from lichens will be possible through the application of new methodological approaches, which involve imitating natural habitat conditions. This will require replacing glucose, sucrose, tryptones and starch with alternative carbon and nitrogen sources, such as amino acids, polyols and heteropolysaccharides.

## Figures and Tables

**Table 1:** Sampling site information and collected lichen species.

Sampling Places	Coordinates	Sample ID	Species
Massif Khibiny. Botanical Cirque. Slope of scree. Tundra belt. Boulder. Rock. ±0 cm below snow level	67°38′46″ N 33°38′55″ E	Ctr_Is-KH	*Cetraria islandica* (L.) Ach.
	67°38′47″ N 33°38′55″ E	N_Niv-KH	*Nephromopsis nivalis* (L.) Divakar, A.Crespo & Lumbsch (syn. *Flavocetraria nivalis* (L.) Kärnefelt & A.Thell)
Massif Khibiny. Botanical Cirque. Moraine slope. Border of birch forest. Soil. 20 cm below snow level		C_St-KH	*Cladonia stellaris* (Opiz) Pouzar & Vĕzda
Massif Khibiny. Slope of Botanical Cirque. Tundra belt. Rock vertical wall in ice film. Above snow level ~100 cm	67°38′31″ N 33°39′19″ E	S_Ves-KH	*Stereocaulon vesuvianum* Pers.
Nenets Autonomous Okrug, Iskateley settlement, Layavozhskaya road	67°39′22″ N 53°8′25″ E	Ctr_Is-NM	*Cetraria islandica* (L.) Ach.
		N_Niv-NM	*Nephromopsis nivalis* (L.) Divakar, A.Crespo & Lumbsch
		C_Arb-NM	*Cladonia arbuscula* (Wallr.) Flot.
		S_Pas-NM	*Stereocaulon paschale* (L.) Hoffm.

**Table 2:** Correspondence between taxonomic rank and percentage identity [45].

Taxonomic Level of Identification	Percentage of Similarity
Species definitively identified	>98%
New species within a genus	94.3–98%
New genus within a family	88.5–94.2%
New family within an order	81.2–88.4%
New order within a class	80.9–81.1%
New class within a phylum	<80.9%

**Table 3:** Identifying lichens using ITS1 gene sequencing data.

Sample ID	Otu Number	GenBank No. of Sequence	Otu Abundance (med.), % *	Query Cover, %	Identity, %	Genbank No. of a Nearest Sequence
Ctr_Is_KH Ctr_Is_NM	Otu3	PX502201	92 27	94	100	MG250320
Ctr_Is_KH Ctr_Is_NM	Otu17	PX502207	1.5 57	92	99.57	KY764999
N_Niv_KH N_Niv_NM	Otu232	PX502209	66.5 65	100	99.6	GU067707
N_Niv_KH N_Niv_NM	Otu1	PX502199	30 30	94	100	MG461626
C_St_KH	Otu2	PX502200	81.5	77	100	MK812280
C_Arb_NM	Otu6	PX502203	27.6	98	99.59	OL694693
	Otu7	PX502204	27.5	91	99.56	MK508932
	Otu9	PX502205	23.8	98	99.59	KY119382
S_Ves_KH	Otu4	PX502202	82.5	91	98.25	LC742699
S_Pas_NM	Otu25	PX502208	57	94	100	HQ650690
	Otu12	PX502206	33	95	100	MT925689

*—averaged over two repetitions.

**Table 4:** The values shown are for the number of final reads, the number of reads in OTUs, the richness of unique taxonomic units, and the mapping percentage of the lichen samples studied.

Sample ID	Reads Initial	Reads in OTU	Richness	Mapped Percent
Ctr_Is_KH	15,353 ± 1394	13,938 ± 986	123 ± 0	90.95 ± 1.84
N_Niv_KH	28,561 ± 3294	27,264 ± 3091	120 ± 11	95.48 ± 0.19
N_Niv_KH (processed) *	28,698 ± 2102	27,614 ± 2086	40 ± 2.5	96.21 ± 0.22
C_St_KH	27,784 ± 3024	26,155 ± 2674	256 ± 24	94.21 ± 0.62
S_Ves_KH	15,340 ± 758	13,348 ± 746	77 ± 26	87.00 ± 0.56
Ctr_Is_NM	8766 ± 2936	7775 ± 2596	102 ± 9	88.72 ± 0.10
N_Niv_NM	28,933 ± 1806	27,368 ± 1820	139 ± 22	94.56 ± 0.38
N_Niv_NM (processed) *	19,160 ± 415	18,316 ± 406	80 ± 18	95.60 ± 0.05
C_Arb_NM	10,290 ± 1709	9053 ± 1540	186 ± 23	87.92 ± 0.36
S_Pas_NM	19,976 ± 216	13,770 ± 1020	172 ± 1	69.00 ± 5.86

*—samples were processed with an abrasive and then washed.

**Table 5:** The mass proportion of mycobiont OTUs and other fungal OTUs in the mycobiome of the studied lichen samples.

Sample ID	OTU, % Mycobiont	OTU, % Others
Ctr_Is_KH	93.44 ± 0.86	6.56 ± 0.86
N_Niv_KH	95.84 ± 1.44	4.16 ± 1.44
N_Niv_KH (processed) *	98.98 ± 0.31	1.02 ± 0.31
C_St_KH	81.17 ± 3.6	18.83 ± 3.60
S_Ves_KH	96.18 ± 2.84	3.82 ± 2.84
Ctr_Is_NM	83.91 ± 1.06	16.09 ± 1.06
N_Niv_NM	94.68 ± 1.17	5.32 ± 1.17
N_Niv_NM (processed) *	96.35 ± 0.15	2.76 ± 0.68
C_Arb_NM	80.70 ± 0.12	19.31 ± 0.12
S_Pas_NM	91.96 ± 0.98	8.04 ± 0.98

*—samples were processed with an abrasive and then washed.

**Table 6:** Species richness and the Shannon and Simpson indices, calculated based on the number and diversity of cultivated fungal taxa from lichens.

Sample ID	Richness	Shannon Index	Simpson Index
Ctr_Is_KH	10	2.24	0.13
Ctr_Is_NM	15	2.82	0.29
N_Niv_KH	5	1.73	0.17
N_Niv_NM	2	0.87	0.50
S_Ves_KH	4	1.06	0.54
S_Pas_NM	5	1.67	0.21
C_Arb_NM	9	2.30	0.15

## Data Availability

The OTU sequences obtained by NGS profiling have been deposited in GenBank under the accession numbers PX406301-PX406485 and PX502199-PX502209. The 18S rRNA (partial), ITS1, 5.8S rRNA, ITS2 and 28S rRNA (partial) gene sequences of pure cultures are deposited in GenBank under the following accession numbers: PX352608-PX352694.

## Supplementary Materials

The following supporting information can be downloaded at: https://www.mdpi.com/article/10.3390/jof11120848/s1, Figure S1. The graphs show the representation values of individual OTUs in the total OTUs array, as well as the statistical error when averaged. Figure S2. The phylogenetic tree shows the distance among unidentified OTUs that were retrieved from two species of lichens, N. nivalis (N_Niv-KH) and S. vesuvianum (S_Ves_KH). The evolutionary history was inferred by using the Maximum Likelihood method and Jukes-Cantor model. The tree with the highest log likelihood (−5242.00) is shown. The percentage of trees in which the associated taxa clustered together is shown next to the branches. Initial tree(s) for the heuristic search were obtained automatically by applying Neighbor-Join and BioNJ algorithms to a matrix of pairwise distances estimated using the Maximum Composite Likelihood (MCL) approach, and then selecting the topology with superior log likelihood value. The tree is drawn to scale, with branch lengths measured in the number of substitutions per site. This analysis involved 33 nucleotide sequences. There were a total of 441 positions in the final dataset. Evolutionary analyses were conducted in MEGA X. Figure S3. Venn diagrams illustrating the presence of shared OTUs in lichens collected from Khibiny (KH) and Naryan-Mar (NM). Shared OTUs are shown in bold. The following lichens are represented: Ctr_Isl (*Cetraria islandica*); N_Niv (*Nephromopsis nivalis*); C_St (*Cladonia stellaris*); C_Arb (*Cladonia arbuscula*); S_Ves (*Stereocaulon vesuvianum*); and S_Pas (*Stereocaulon paschale*). Figure S4. The Venn diagrams illustrate the genera that are shared by lichens collected from Khibiny (A) and Naryan-Mar (B). The shared genera are shown in bold.Table S1. The list of the genera and species that were identified based on an analysis of the ITS1 gene sequence, compared with sequences of this gene from the MycoBank and NCBI databases. Ctr_Is, *Cetraria islandica*; N_Niv, *Nephromopsis nivalis*; C_St, *Cladonia stellaris*; C-Arb, *C. arbuscula*; S_Ves, *Stereocaulon vesuvianum*; S_Pas, *S. paschale*. KH, Khibiny; NM, Naryan-Mar. Table S2. A list of the strains isolated from the six lichen species in the Khibiny and Naryan-Mar regions. UP, in the process of depositing. Table S3. Taxonomic affiliation and the relative contribution of OTUs to the mycobiome communities of the lichens studied.

## Abbreviations

The following abbreviations are used in this manuscript:

OTU	Operational Taxonomic Unit
PBS	Phosphate-Saline Buffer
CTAB	Cetyltrimethylammonium Bromide
BLAST	Basic Local Alignment Search Tool
KPABG	Kola Polar Alpine Botanical Garden
PABGI KSC RAS	Polar-Alpine Botanical Garden-Institute of the Kola Science Center of the Russian Academy of Sciences
CFU	Colony-Forming Units
