# Supplementary material for: Mycobiomes of Six Lichen Species from the Russian Subarctic: A Culture-Independent Analysis and Cultivation Study
Source: J Fungi (Basel). 2025 Nov 29;11(12):848. doi: 10.3390/jof11120848 (PMC12733807; doi:10.3390/jof11120848)
Supplement: Supplementary file 1 [file jof-11-00848-s001.zip › SupplementaryFigures.pdf]

Mycobiomes of Six Lichen Species from the Russian Subarctic: A Culture-Independent Analysis and Cultivation Study

Armen Hakobjanyan[1,2], Alexey Melekhin[3,4], Marina Sukhacheva[5], Alexey Beletsky[5], Timofey Pankratov[1] *

[1] *S.N. Winogradsky Institute of Microbiology, Research Centre of Biotechnology of RAS, 119071, Moscow, Russia*

[2] *National Research University "Higher school of economics", 101000, Moscow, Russia*

[3] *N.A. Avrorin Polar-Alpine Botanical Garden Institute, 184209, Apatity, Russia*

[4] *Tobolsk complex scientific station of the Ural Branch of RAS, 626152, Tobolsk, Russia*

[5] *Skryabin Institute of Bioengineering, Research Centre of Biotechnology of RAS, 119071, Moscow, Russia*

* Corresponding author: t.pankratov@inmi.ru or tpankratov@gmail.com

Supplementary Figures

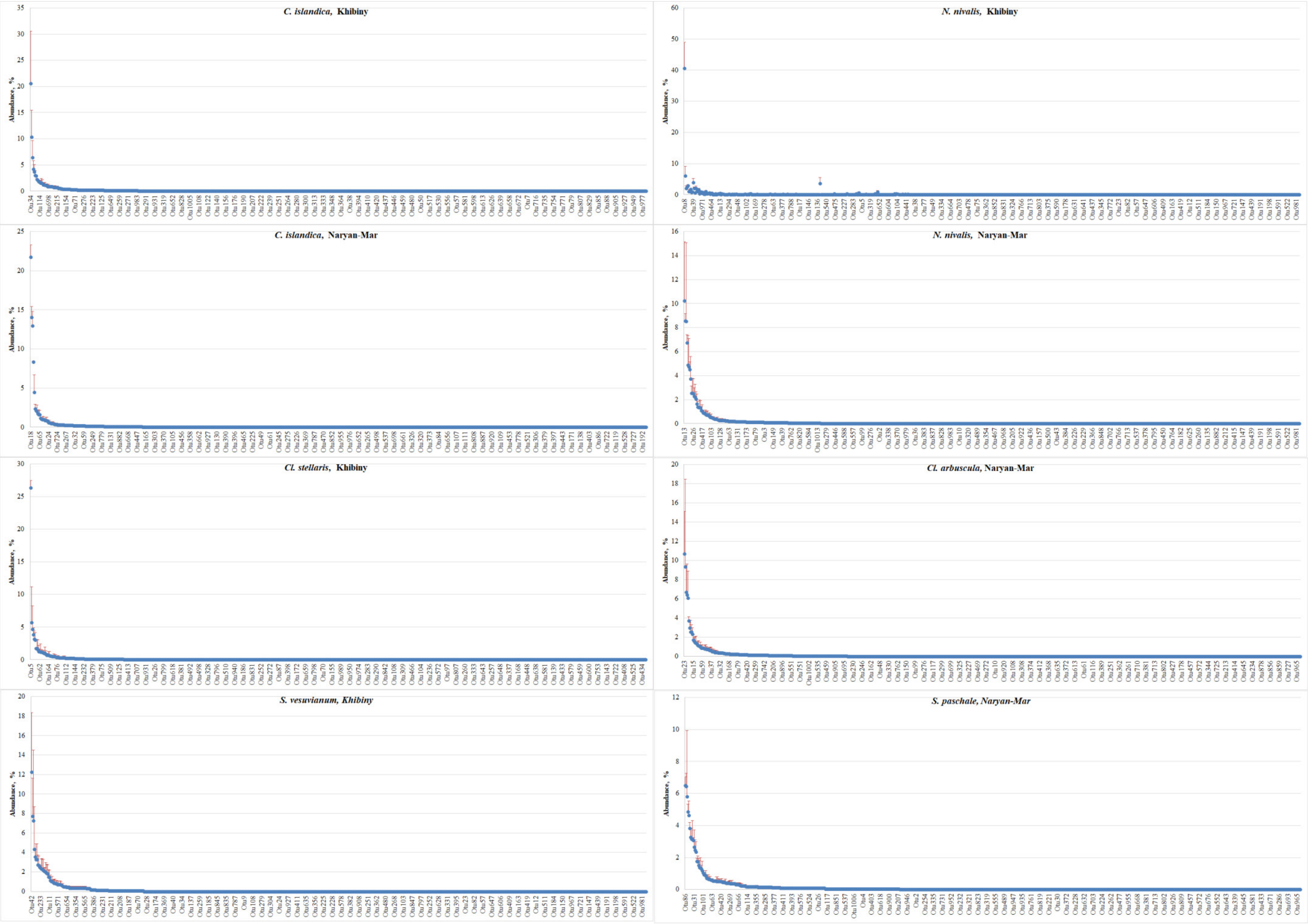

Supplementary Figure S1. The graphs show the representation values of individual OTUs in the total OTUs array, as well as the statistical error when averaged.

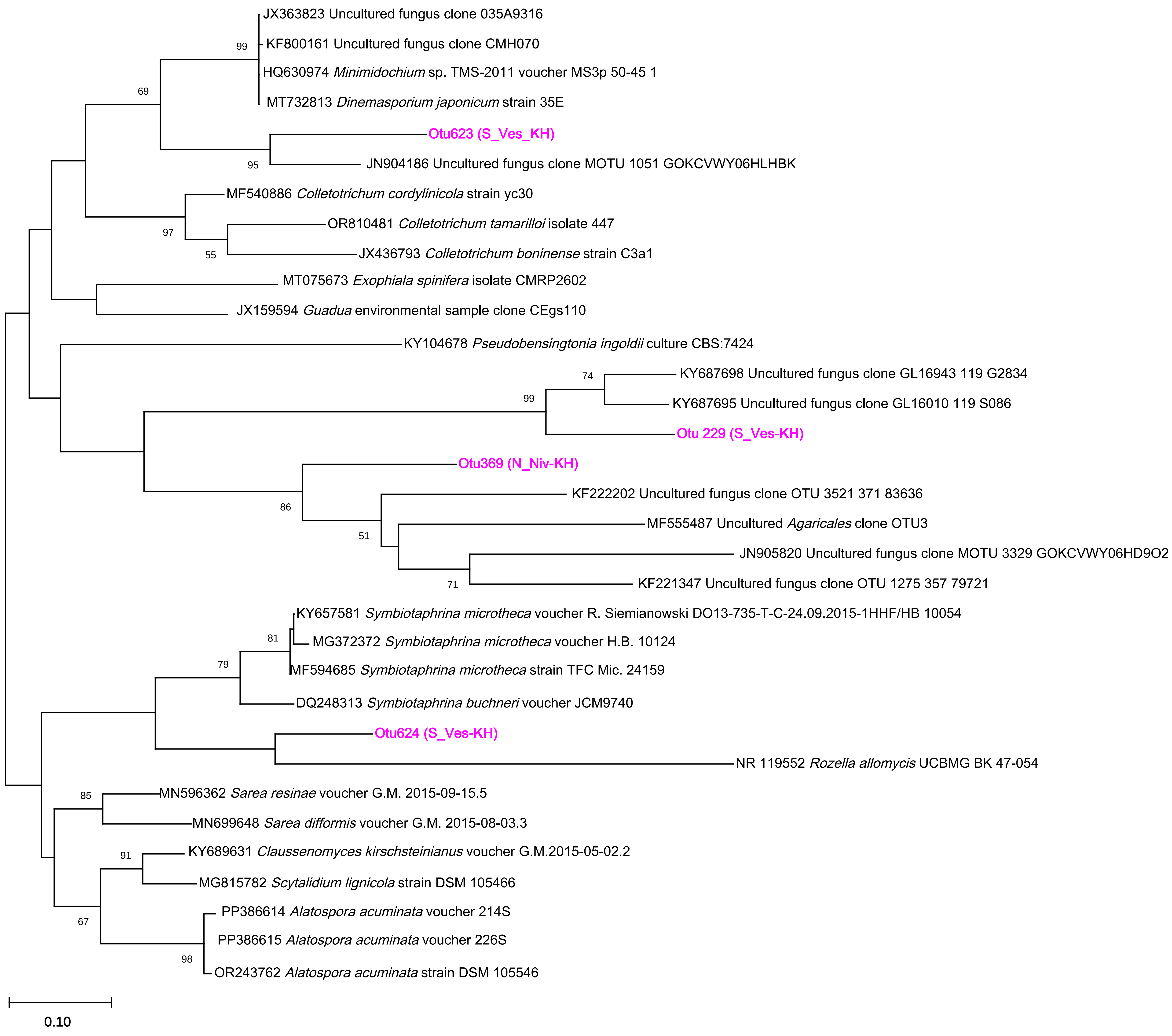

Supplementary Figure S2. The phylogenetic tree shows the distance among unidentified OTUs that were retrieved from two species of lichens, *N. nivalis* (N_Niv-KH) and *S. vesuvianum* (S_Ves_KH). The evolutionary history was inferred by using the Maximum Likelihood method and Jukes-Cantor model. The tree with the highest log likelihood (-5242.00) is shown. The percentage of trees in which the associated taxa clustered together is shown next to the branches. Initial tree(s) for the heuristic search were obtained automatically by applying Neighbor-Join and BioNJ algorithms to a matrix of pairwise distances estimated using the Maximum Composite Likelihood (MCL) approach, and then selecting the topology with superior log likelihood value. The tree is drawn to scale, with branch lengths measured in the number of substitutions per site. This analysis involved 33 nucleotide sequences. There were a total of 441 positions in the final dataset. Evolutionary analyses were conducted in MEGA X.

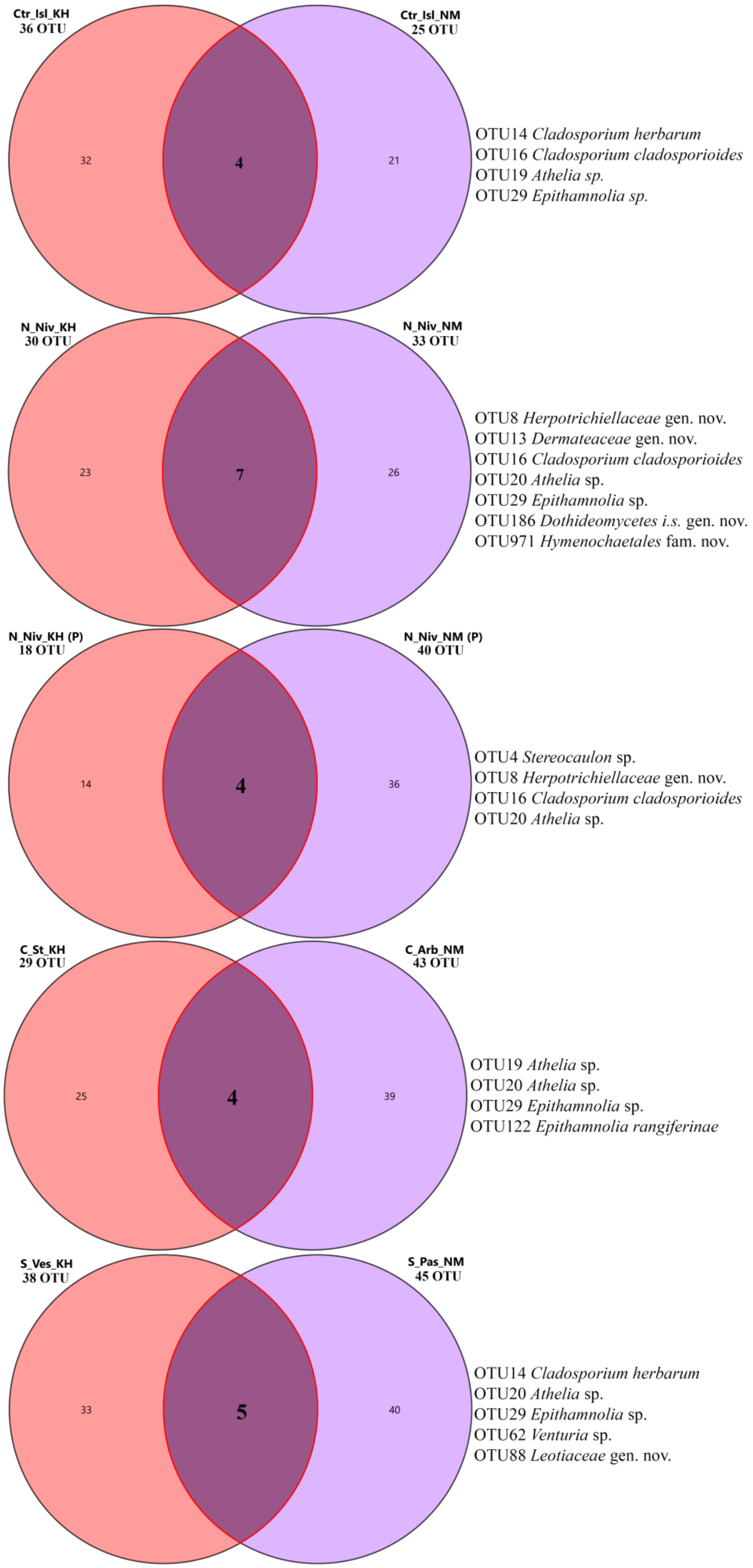

Supplementary Figure S3. Venn diagrams illustrating the presence of shared OTUs in lichens collected from Khibiny (KH) and Naryan-Mar (NM). Shared OTUs are shown in bold. The following lichens are represented: Ctr_Isl (*Cetraria islandica*); N_Niv (*Nephromopsis nivalis*); C_St (*Cladonia stellaris*); C_Arb (*Cladonia arbuscula*); S_Ves (*Stereocaulon vesuvianum*); and S_Pas (*Stereocaulon paschale*).

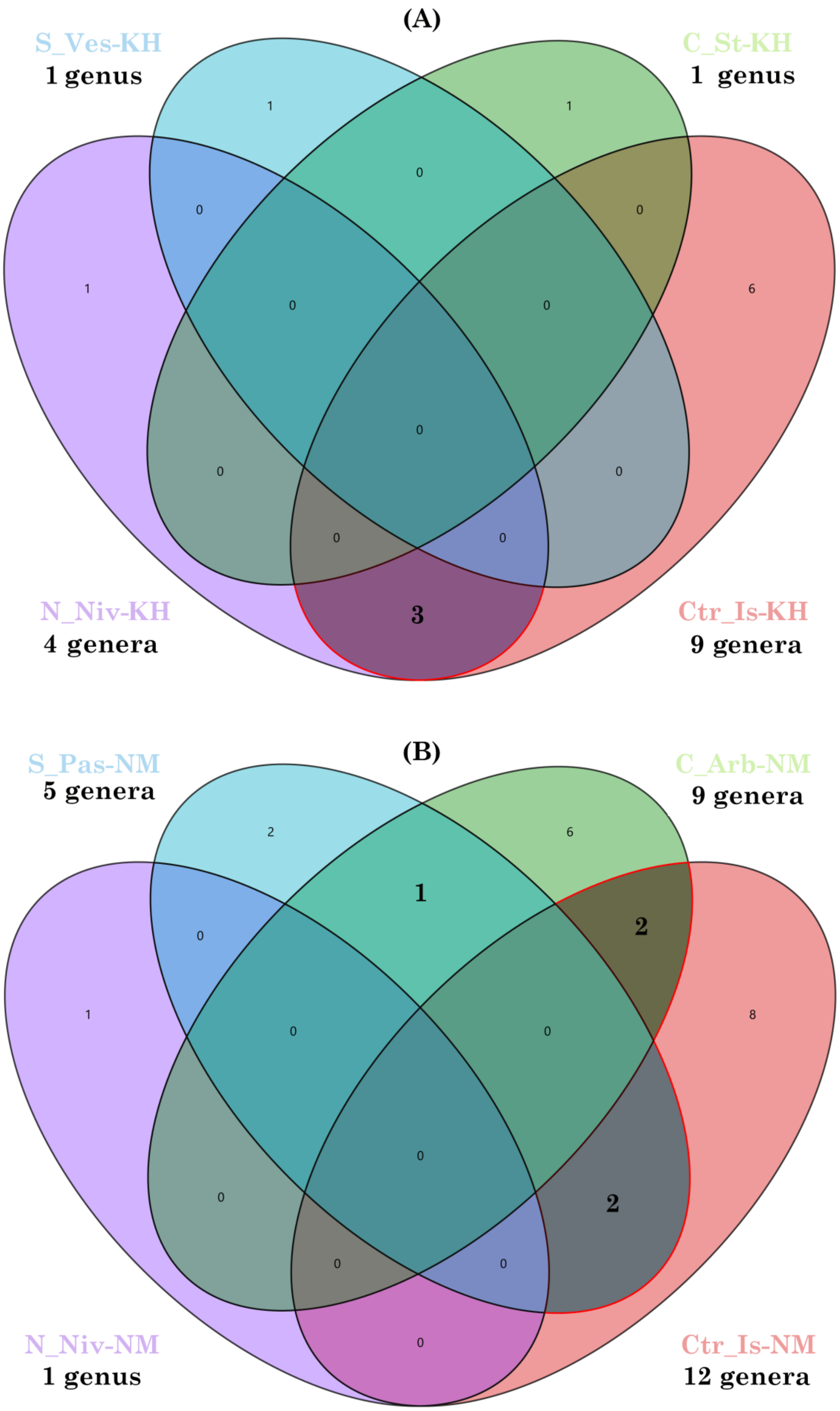

Supplementary Figure S4. The Venn diagrams illustrate the genera that are shared by lichens collected from Khibiny (A) and Naryan-Mar (B). The shared genera are shown in bold.
