# Supplementary material for: Mycobiomes of Six Lichen Species from the Russian Subarctic: A Culture-Independent Analysis and Cultivation Study
Source: J Fungi (Basel). 2025 Nov 29;11(12):848. doi: 10.3390/jof11120848 (PMC12733807; doi:10.3390/jof11120848)
Supplement: Supplementary file 1 [file jof-11-00848-s001.zip › SupplementaryTables_1-2.pdf]

Mycobiomes of Six Lichen Species from the Russian Subarctic: A Culture-Independent Analysis and Cultivation Study

Armen Hakobjanyan[1,2], Alexey Melekhin[3,4], Marina Sukhacheva[5], Alexey Beletsky[5], Timofey Pankratov[1] *

[1] *S.N. Winogradsky Institute of Microbiology, Research Centre of Biotechnology of RAS, 119071, Moscow, Russia*

[2] *National Research University "Higher school of economics", 101000, Moscow, Russia*

[3] *N.A. Avrorin Polar-Alpine Botanical Garden Institute, 184209, Apatity, Russia*

[4] *Tobolsk complex scientific station of the Ural Branch of RAS, 626152, Tobolsk, Russia*

[5] *Skryabin Institute of Bioengineering, Research Centre of Biotechnology of RAS, 119071, Moscow, Russia*

* Corresponding author: t.pankratov@inmi.ru or tpankratov@gmail.com

Supplementary Tables

Supplementary Table S1. The list of the genera and species that were identified based on an analysis of the ITS1 gene sequence, compared with sequences of this gene from the MycoBank and NCBI databases. Ctr_Is, *Cetraria islandica*; N_Niv, *Nephromopsis nivalis*; C_St, *Cladonia stellaris*; C-Arb, *C. arbuscula*; S_Ves, *Stereocaulon vesuvianum*; S_Pas, *S. paschale.* KH, Khibiny; NM, Naryan-Mar.

Sample ID	Genera	Species
**Ctr_Is-KH**	*Carbonea, Cladonia, Cladophialophora, Cladosporium, Cortinarius, Epithamnolia, Fibularhizoctonia (Athelia), Hypholoma, Infundichalara, Leptosporomyces, Lichenoconium, Sorocybe, Stereocaulon, Trechispora*	*Infundichalara microchona, Cladosporium hillianum, Trechispora byssinella, Cladosporium cladosporioides, Cortinarius armillatus, Hypholoma capnoides*
**Ctr_Is-NM**	*Alternaria, Cladonia, Cladophialophora, Cladosporium, Epithamnolia, Fibularhizoctonia (Athelia), Nothophoma, Ochrolechia*	*Cladosporium cladosporioides, Cladophialophora minutissima, Cladosporium hillianum*
**N_Niv-KH**	*Athelia, Burgellopsis, Cladosporium, Epithamnolia, Hypholoma, Hypomyces,Leptosporomyces, Ochrolechia, Penidiella, Trechispora*	*Cladosporium hillianum, Burgellopsis nivea, Trechispora byssinella, Ochrolechia mahluensis, Hypholoma capnoides*
**N_Niv-NM**	*Athelia, Aureobasidium, Bergerella, Ciliolarina, Cladonia, Cladosporium,Coccomyces, Epithamnolia, Pseudoteratosphaeria, Readeriella, Rosellinia*	*Cladonia gracilis, Cladosporium angustiherbarum, Epithamnolia rangiferinae, Cladosporium hillianum, Aureobasidium proteae, Epithamnolia xanthoriae*
**C_St-KH**	*Coleophoma, Epithamnolia, Fibularhizoctonia, Glutinomyces, Mortierella, Mrakia, Venturia*	*Venturia minuta, Epithamnolia rangiferinae*
**C_Arb-NM**	*Apioperdon, Athelia, Aureobasidium, Cladonia, Cladophialophora, Cladosporium, Epithamnolia, Fibularhizoctonia, Hormonema, Hyaloscypha, Hyphodiscus, Nephromopsis, Nothophoma, Penidiella, Readeriella*	*Cladosporium cladosporioides, Cladonia subulata, Aureobasidium pullulans, Hyaloscypha hepaticicola, Apioperdon pyriforme*
**S_Ves-KH**	*Athelia, Cladosporium, Cortinarius, Epithamnolia, Leptosporomyces, Mrakia, Tolypocladium, Trichoderma, Umbilicaria, Venturia*	*Umbilicaria vellea, Cortinarius armillatus, Trichoderma viride, Tolypocladium inflatum, Cladosporium herbarum*
**S_Pas-NM**	*Apioperdon, Athelia, Aureobasidium, Baeomyces, Cladonia, Cladophialophora, Cladosporium, Coniothyrium, Epithamnolia, Glutinomyces, Hormonema, Hyaloscypha, Oidiodendron, Penicillium, Penidiella, Readeriella, Venturia*	*Cladonia gracilis, Aureobasidium pullulans, Cladosporium herbarum, Coniothyrium lignorum, Cladosporium cladosporioides, Hyaloscypha hepaticicola, Cladonia subulata, Cladophialophora minutissima Penicillium yezoense, Apioperdon pyriforme, Baeomyces rufus, Epithamnolia xanthoriae*
**N_Niv-KH (P)**	*Athelia, Cladosporium, Cortinarius, Epithamnolia*	The species has not been identified
**N_Niv-NM (P)**	*Allantophomopsis, Apioperdon, Athelia, Aureobasidium, Bergerella, Cladonia, Cladosporium, Epithamnolia, Lichenoconium, Neophaeomoniella, Phaeotheca, Pseudoteratosphaeria, Sarcinomyces, Stereocaulon*	*Cladosporium hillianum, Cladosporium angustiherbarum, Aureobasidium proteae, Apioperdon pyriforme, Epithamnolia rangiferinae*

Supplementary Table S2. A list of the strains isolated from the six lichen species in the Khibiny and Naryan-Mar regions. UP, in the process of depositing.

Lichen species	Strain	GenBank number	Organism	GenBank number	Query Cover	Per. Ident
***Cetraria islandica* (Khibiny)**	1.1.1.1	PX352609	*Hebeloma angustilamellatum*	AY575919	100	90.15
	1.1.1.2	UP	*Melanomma pulvis-pyrius*	KY189979	100	99.36
	1.1.1.4	PX352616	*Leptosporomyces galzinii*	EU118642	61	95.54
	1.1.2.1	PX352614	*Cladosporium cladosporioides*	OR243761	100	99.54
	1.1.2.2	PX352612	*Hypoxylon fragiforme*	KU684022	99	97.61
	1.1.2.3	PX352610	*Hypholoma capnoides*	KC176278	100	98.73
	1.1.2.P2	UP	*Cordyceps farinosa*	AB027379	100	95.66
	1.2.1.1	PX352613	*Occultifur cerinomycicola*	PP908454	100	88.44
	1.2.1.3	UP/checking	*Occultifur wangii*	MN128424	91	91.18
	1.2.1.5	PX352608	*Tremella shuangheensis*	MK050285	100	85.49
	1.2.1.6	UP/checking	*Phaeothecoidea eucalypti*	EU019280	97	98.12
	1.2.2.4	PX352611	*Myriangium duriaei*	OM238138	99	89.17
	1.2.2.6	PX352615	*Lichenoconium erodens*	HQ174267	94	98.63
***Nephromopsis nivalis* (Khibiny)**	2.1.1.1	UP	*Cladophialophora sylvestris*	EU035413	94	95.41
	2.1.2.1	PX352621	*Hypholoma capnoides isolate T-773*	KC176278	90	98
	2.1.2.2	PX352617	*Myriangium duriaei*	OM238138	90	92.60
	2.1.2.3	PX352619	*Lichenoconium aeruginosum*	MH876629	52	97.18
	2.1.2.4	PX352618	*Leptosporomyces galzinii*	EU118642	81	99.11
	2.1.2.5	PX352620	*Lichenoconium aeruginosum*	HQ174269	61	96.39
	2.1.2.6	PX352622	*Cladophialophora minutissima*	LC085208	93	94.88
***Cladonia stellaris* (Khibiny)**	3.3.1.1	PX352624	*Venturia polygoni-vivipari*	EU035466	100	97.99
	3.3.1.2	PX352625	*Byssocorticium caeruleum*	GQ162814	100	89.72

Supplementary Table S2: (continued).

Lichen species	Strain	GenBank number	Organism	GenBank number	Query Cover	Per. Ident
***Stereocaulon vesuvianum* (Khibiny)**	4.10^3.1.1	PX352628	*Fayodia gracilipes*	KC176299	100	92.13
	4.10^3.1.2	PX352632	*Tolypocladium inflatum*	AB044645	100	96.51
	4.10^3.1.4	PX352635	*Tolypocladium inflatum*	AB044645	100	96.22
	4.10^3.1.5	PX352629	*Trimmatostroma betulinum*	EU019299	100	98.87
	4.10^3.1.6	PX352626	*Tolypocladium inflatum*	AB044645	100	97.00
	4.2.2.1	PX352634	*Tolypocladium inflatum*	AB044645	100	97.18
	4.2.2.10	UP	*Hyphodiscus brachyconius*	GU727557	66	89.86
	4.2.2.12	PX352630	*Tolypocladium inflatum*	AB044645	99	95.90
	4.2.2.2	PX352636	*Tolypocladium inflatum*	AB044645	100	97.33
	4.2.2.3	PX352640	*Tolypocladium inflatum*	AB044645	100	96.89
	4.2.2.5	PX352637	*Tolypocladium inflatum*	AB044645	100	99.36
	4.2.2.6	PX352639	*Tolypocladium inflatum*	AB044645	100	99.45
	4.2.2.7	PX352638	*Tolypocladium inflatum*	AB044645	100	97.06
	4.2.2.8	PX352631	*Tolypocladium inflatum*	AB044645	100	96.80
	4.2.2.9	PX352633	*Tolypocladium inflatum*	AB044645	100	95.72
	4.3.2.3	PX352627	*Tolypocladium bacillisporum*	LC684522	100	95.51

Supplementary Table S2: (continued).

Lichen species	Strain	GenBank number	Organism	GenBank number	Query Cover	Per. Ident
Cetraria islandica (Naryan-Mar)	5.1.1.1	PX352643	*Phoma herbarum*	MF120206	96	99.80
	5.1.1.2	PX352645	*Ascochyta phacae*	EU167570	100	99.16
	5.1.1.3	PX352663	*Vishniacozyma pseudopenaeus*	MK050333	92	94.05
	5.1.1.4	PX352654	*Microsphaeropsis olivacea*	MH871969	100	99.62
	5.2.1.1	PX352660	*Sydowia polyspora*	ON193835	100	99.19
	5.2.1.11	PX352662	*Occultifur cladoniae*	MN128423	99	98.89
	5.2.1.12	PX352666	*Myriangium hispanicum*	MH855426	97	84.84
	5.2.1.13	PX352648	*Rhodotorula sp. strain KBP:Y-7883*	PV738092	97	99.41
	5.2.1.14	PX352644	*Capronia villosa*	AF050261	98	90.51
	5.2.1.2	PX352661	*Oidiodendron truncatum*	KF835845	80	96.85
	5.2.1.3	PX352665	*Ballistosporomyces changbaiensis*	KP020105	75	83.25
	5.2.1.4	PX352653	*Catenulifera brachyconia*	GU727557	100	96.06
	5.2.1.5	PX352652	*Neocatenulostroma microsporum*	EU167572	100	91.57
	5.2.1.6	PX352649	*Knufia epidermidis*	LC414357	75	94.94
	5.2.1.7	PX352642	*Hyalodendriella betulae*	EU040232	96	91.54
	5.2.1.8	PX352650	*Scytalidium circinatum*	MH862195	100	90.15
	5.2.2.1	PX352647	*Metapochonia bulbillosa*	LN901142	100	96.47
	5.2.2.5	PX352641	*Cladophialophora minutissima*	LC085208	100	98.23
	5.2.2.6	PX352667	*Oidiodendron maius*	MT321755	100	93.33
	5.2.2.7	PX352646	*Sakaguchia lamellibrachiae*	AB025999	99	89.85
	5.2.2.8	PX352656	*Oidiodendron maius*	MT321755	100	93.78
	5.3.1.3	PX352655	*Rhizoplaca parilis*	MN756808	92	87.11
	5.3.1.4	PX352668	*Rhizoplaca parilis*	MN756808	90	87.05
	5.3.2.1	PX352651	*Hyalodendriella betulae*	EU040232	100	90.06
	5.3.2.2	PX352657	*Neohortaea acidophila*	OL739260	100	90.39
	5.3.2.3	PX352659	*Cladophialophora minutissima*	LC085208	97	98.15
	5.3.2.7	PX352664	*Rhizoplaca parilis*	MN756808	92	87.41
	5.3.2.8	PX352658	*Rhizoplaca parilis*	MN756808	92	87.20

Supplementary Table S2: (continued).

Lichen species	Strain	GenBank number	Organism	GenBank number	Query Cover	Per. Ident
Nephromopsis nivalis (Naryan-Mar)	6.2.1.1	PX352670	*Tolypocladium inflatum*	JF796050	97	94.51
	6.2.1.3	PX352669	*Ballistosporomyces changbaiensis*	KP020105	100	87.29
Cladonia arbuscula (Naryan-Mar)	7.1.2.1	PX352673	*Cadophora fastigiata*	MF494623	100	98.21
	7.1.2.10	PX352677	*Oidiodendron tenuissimum*	PV835072	94	98.57
	7.1.2.11	PX352681	*Occultifur cladoniae*	MN128423	97	96.51
	7.1.2.12	PX352671	*Vishniacozyma carnescens*	LC203729	100	98.43
	7.1.2.2	PX352678	*Cladosporium cladosporioides*	KX664389	100	99.86
	7.1.2.3	PX352672	*Aureobasidium pullulans*	ON193740	100	98.98
	7.1.2.4	PX352679	*Thelebolus microsporus*	MF120208	100	99.35
	7.1.2.5	PX352680	*Cadophora sp. CJL-2014 strain Rs-R-38*	KJ542331	100	98.18
	7.1.2.6	PX352682	*Protoventuria alpina*	EU035444	100	95.71
	7.1.2.7	PX352674	*Cladosporium ossifragi*	EF679381	96	98.44
	7.1.2.8	PX352676	*Cladosporium ossifragi*	EF679381	98	99.44
	7.1.2.9	PX352675	*Filobasidium magnum*	JX188126	100	99.50
Stereocaulon paschale (Naryan-Mar)	8.2.1.1	PX352691	*Phoma herbarum*	MF120206	100	99.73
	8.2.1.11	PX352694	*Vishniacozyma victoriae*	KY105835	100	99.51
	8.2.1.13	PX352690	*Phoma herbarum*	MF120206	100	99.63
	8.2.1.14	PX352689	*Penicillium lividum*	AF033406	99	99.46
	8.2.1.2	PX352692	*Beauveria brongniartii*	AB027381	99	98.48
	8.2.1.3	PX352684	*Penicillium lividum*	AF033406	99	98.19
	8.2.1.4	PX352687	*Penicillium lividum*	AF033406	100	98.56
	8.2.1.6	PX352688	*Beauveria brongniartii*	AB027381	100	98.54
	8.2.1.7	PX352693	*Phoma herbarum*	MF120206	100	98.15
	8.2.1.8	PX352683	*Phoma herbarum*	MF120206	99	99.35
	8.2.1.9	PX352686	*Sydowia polyspora*	ON193835	100	97.70
	8.3.2.2	PX352685	*Venturia hystrioides*	EU035459	90	92.78
